# Recent speciation associated with range expansion and a shift to self-fertilization in North American *Arabidopsis*

**DOI:** 10.1038/s41467-022-35368-1

**Published:** 2022-12-08

**Authors:** Yvonne Willi, Kay Lucek, Olivier Bachmann, Nora Walden

**Affiliations:** 1 Department of Environmental Sciences, University of Basel, Basel, Switzerland; 2 Department of Plant Sciences, University of Wageningen, Wageningen, The Netherlands; 3 Centre for Organismal Studies, University of Heidelberg, Heidelberg, Germany

**Keywords:** Speciation, Population genetics, Plant evolution, Evolutionary ecology

## Abstract

The main processes classically evoked for promoting reproductive isolation and speciation are geographic separation reducing gene flow among populations, divergent selection, and chance genomic change. In a case study, we present evidence that the additional factors of climate change, range expansion and a shift in mating towards inbreeding can initiate the processes leading to parapatric speciation. At the end of the last Pleistocene glaciation cycle, the North American plant *Arabidopsis lyrata* expanded its range and concomitantly lost its reproductive mode of outcrossing multiple times. We show that in one of the newly colonized areas, the self-fertilizing recolonization lineage of *A. lyrata* gave rise to selfing *A. arenicola*, which expanded its range to subarctic and arctic Canada and Greenland, while the parental species remained restricted to temperate North America. Despite the vast range expansion by the new species, mutational load did not increase, probably because of selfing and quasi-clonal selection. We conclude that such peripheral parapatric speciation combined with range expansion and inbreeding may be an important but so far overlooked mode of speciation.

## Abstract (web summary)

Parapatric speciation occurs when reproductive isolation arises without full geographic isolation. Here, the authors combine genomic and phylogeographic analyses to illustrate a case of parapatric speciation attributed to climate change, range expansion and mating system shift.

## Introduction

New species arise by the evolution of reproductive barriers. How these evolve was typically categorized by the underlying geographic scenario^1^: The geographic spectrum ranges from allopatric to sympatric speciation, with species splitting either in total geographic isolation or from a freely interbreeding population, respectively. Parapatric speciation takes an intermediate position, with gene flow being partially restricted over space. However, more dichotomies need to be considered for understanding patterns and processes in speciation^2^. The process of speciation may be better described by the sequence of progression along axes of spatial, ecological and mating differentiation, accommodating that the evolution of reproductive barriers can be diverse and dynamic^2^. Furthermore, interactions among processes and the type of genetic change they cause are relevant^3–6^. Here we present a case of parapatric speciation that emphasizes the interplay between three processes which are common but to our knowledge have not yet been considered for their combined impact on speciation: climate change, range expansion, and mating-system evolution.

Periods of strong climate change have been linked to major extinction events both on a regional and a global scale^7–9^, but also to giving rise to new species and clades^9,10^. Relatively recently, the Pleistocene glaciation cycles contributed to repeated retractions of many species, particularly of temperate zones, to glacial refugia^7,11,12^. Because populations of different refugia often diverged through genetic drift or selection, isolation in separate refugia has been considered as initiating or at least promoting the speciation process, if lineages from different refugia coexist after new range expansion^11,13,14^. Here we argue that the expansion process itself may be another driver of speciation. Postglacial range expansions are often associated with a reduction in genetic diversity^11,12^, indicating that they progressed by serial founder events with little gene flow^15^. Founder events favor self-fertilization, formalized by Baker’s rule of mating system shifts from outcrossing to selfing in hermaphroditic organisms due to long-distance dispersal^16^. Therefore, climate change, range expansion with serial founder events coupled with mating shifts to selfing may form a triplet of frequently occurring events over evolutionary time that can initiate and/or promote speciation.

Prevailing theoretical work on speciation with some gene flow is though not too optimistic about its importance for the emergence of new species. Theory typically assumes an environmental gradient, which is likely to exist in many cases of range expansion due to climate change. While theory predicts that adaptive clines can establish along environmental gradients if selection is stronger than migration^17,18^, branching into separate species along such gradients requires assortative mating in sexual organisms^18–20^. Assortative mating combined with small population size—as is typical at expanding range edges—comes with the problem of mutation accumulation likely leading to a backlash in the speciation process because hybrids of the diverging lineages would have a fitness benefit^21^. However, mating system shifts to selfing could provide an evolutionary twist to parapatric speciation: Self-fertilization results in restricted gene flow and the promotion of speciation^22,23^. When combined with larger population size, selfing can furthermore lead to the expression of recessive deleterious mutations in the homozygous state on which purifying selection can act, a process known as purging of the mutational load^24^.

**Fig. 1:**
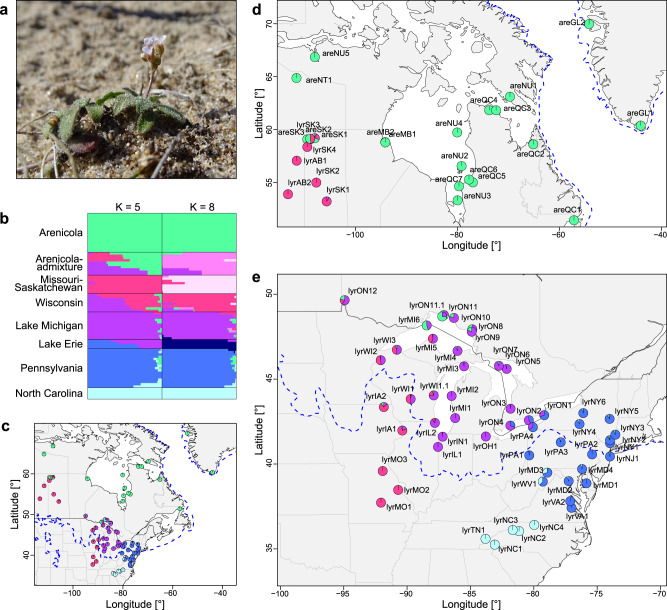
Geography of speciation. North American *Arabidopsis lyrata* (abbreviated with lyr, followed by the abbreviation for state [US] or province [CAN]) and *A. arenicola* (are) were found to be grouped best into five ancestral genetic clusters roughly corresponding to geography (ADMIXTURE results for K = 5). **a** Picture of a particularly hairy *A. arenicola* from Churchill, Manitoba (photo: Yvonne Willi). **b** Cluster assignment under K = 5 and K = 8 (with the second lowest cross validation error) is shown by sorting samples by geography. For populations with multiple individuals, mean cluster assignment is shown. Genetic clusters were named according to their geographic center. **c** Cluster assignment displayed as pie charts under K = 5 is shown on the map of eastern North America. **d** Map of northern populations of *Arabidopsis lyrata* and *A. arenicola* as in panel **c**. **e** Map of the main distribution of *Arabidopsis lyrata* as in panel **c**. The dashed blue line in panels **c**, **d** and **e** represents the maximum extent of the ice sheet during the last glaciation cycle. Source data are provided as a Source data file.

Here we study a likely candidate of parapatric speciation driven by postglacial range expansion and an evolutionary shift to selfing: *Arabidopsis arenicola* (Fig. 1a) and *A.* *lyrata* subsp. *lyrata* of North America^25^. Originally, *A. arenicola* was assigned to the genus *Eutrema* or *Arabis* based on morphology, but genetic analyses positioned it in the genus *Arabidopsis*^26^. Recent genomic verification revealed that *A. arenicola* and North American *A. lyrata* are sister taxa^27,28^. Intrinsic reproductive isolation between the two species is quite strong, with artificial crossing resulting in high failure in fruit and seed set, and in seed germination^29^. Apart from morphology, the species also differ in latitudinal distribution. While *A. arenicola* occurs only in areas that were covered by ice during the last glacial maximum (LGM), i.e., in subarctic and arctic northeastern Canada and on Greenland, *A. lyrata* subsp. *lyrata* is restricted mostly to the temperate eastern and midwestern US, with some extension into southern Ontario and the Prairie Provinces of Canada. Previous research on predominantly outcrossing *A. lyrata* reported range expansion from two LGM refugia, one in the eastern US and one in the Driftless Area of Wisconsin, and that during expansion, shifts to self-compatibility and predominant selfing occurred in several regions that are now at the periphery of the species’ eastern and western distributions^30,31^. Here we test whether *A. arenicola* originated through postglacial parapatric speciation and reconstruct its historical dynamics of range expansion, mating system, demography, and mutational load.

## Results

### Place and date of speciation

**Fig. 2:**
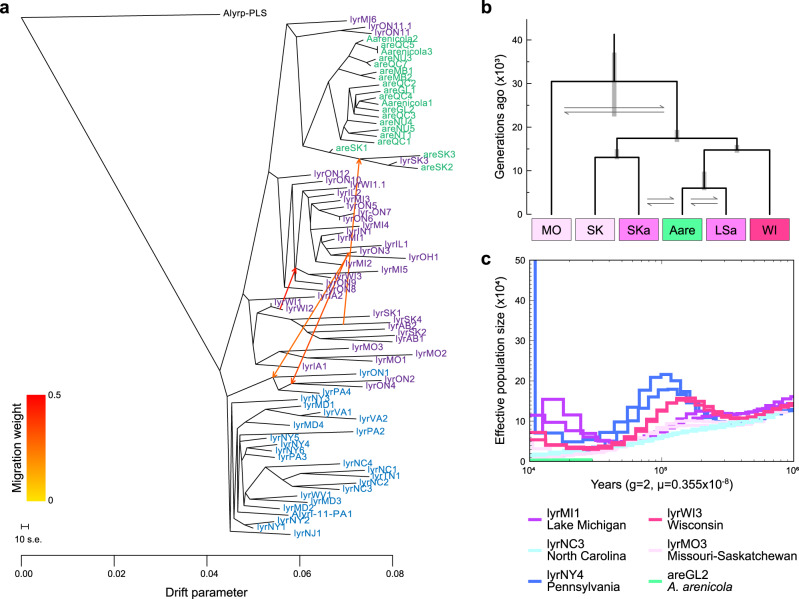
Nuclear phylogeny with admixture, split times, and historic effective population sizes. **a** TreeMix tree based on biallelic synonymous sites of North American *Arabidopsis lyrata* (abbreviated with lyr) and *A. arenicola* (are). Migration events and their inferred direction are displayed as orange arrows (scale given from yellow to red). The scale of 10x the standard error for the drift parameter is indicated. Individual names are colored according to their origin for North American samples (eastern cluster of *A. lyrata* in blue, western cluster in purple [populations of Lake Erie in colors of predominant ADMIXTURE assignment], *A. arenicola* in green). **b** Hierarchical demographic modeling revealed that the best supported model was the one with *A. arenicola* splitting from northwestern Lake Superior *A. lyrata* (LSa) some 6000 generations ago, with earlier splits within *A. lyrata* being those of Wisconsin, then of the Prairie Provinces of Canada (SK, and SKa with signs of admixture), and finally of the Missouri populations. Gray bars for split times indicate 95% confidence intervals. The model considered gene flow, indicated by the gray arrows. **c** Historic effective population size as revealed from sequence analysis based on individuals by PSMC. Results of two individuals of a representative population are shown for each of the five clusters (K = 5), as well as samples from Wisconsin and *A. arenicola*. Population size was estimated using a generation time of two years and mutation rate of 0.355 × 10^−8^. Results for *A. arenicola* indicate low genetic diversity linked to its selfing reproduction. Source data are provided as Source data files.

We analyzed the geographic history of North American *A. lyrata* and *A. arenicola* by using individual whole-genome sequence data from across the entire distribution of both species (Supplementary Data 1) to locate the split between them. To establish dichotomous phylogenetic trees, we excluded individuals that showed evidence of admixture (admixture proportion >5%) based on an a priori cluster analysis (Supplementary Note, Supplementary Fig. 1). A maximum likelihood tree on nuclear single nucleotide polymorphisms (SNPs) supported that the genus *Arabidopsis* colonized North America from Eurasia via Beringia^32^ (Supplementary Note, Supplementary Fig. 2). The split between European and North American *A. lyrata* was dated to 349 kyr and 460 kyr based on nuclear and plastome data, respectively (Supplementary Note, Supplementary Figs. 3–6). The deepest split within North America in the nuclear tree separated western from eastern populations of *A. lyrata*, with *A. arenicola* being a monophyletic group within western *A. lyrata* (splits illustrated based on cluster analysis in Fig. 1c–e; Supplementary Fig. 2). A TreeMix relatedness tree on nuclear allele frequencies, which included populations with signs of admixture and accounted for gene flow, indicated that *A. arenicola* were most closely related to *A. lyrata* populations of northwestern Lake Superior, Isle Royal (MI6) and Terrace Bay (ON11, ON11.1) (Fig. 2a, Supplementary Note, Supplementary Figs. 7–12). These analyses were concordant with *A. arenicola* having emerged from the western clade of *A. lyrata*, and the TreeMix tree suggested that speciation happened in the area of north shore Lake Superior, at the periphery of the distribution of *A. lyrata*. Furthermore, the tree suggested secondary contact in northern Saskatchewan, at Lake Athabasca.

The phylogenomic hypothesis on the origin of the split and its timing were tested by sequential, hierarchical demographic modeling based on site frequency spectra of pooled western *Arabidopsis* super-populations in four steps (Supplementary Fig. 13, Supplementary Data 2–4). Alternative models included *A. arenicola* splitting further west in the Prairie Provinces or being older than the LGM. In a first step, we resolved the relationship among three western backbone super-populations of *A. lyrata* that had shown no evidence of admixture with *A. arenicola*, Missouri (MO), Saskatchewan/Prairie Provinces (SK), and Wisconsin (WI). Comparisons confirmed that Missouri is an old and isolated rear-edge region of the *A. lyrata* distribution. In a second step, we found that the placing of un-admixed *A. arenicola* next to the Wisconsin super-population was better supported than the placing to the Saskatchewan super-population. In the third step, we explored whether splits among the northern populations had occurred generally before or since the LGM, supporting the latter scenario. In the final, fourth step of model comparisons, north shore Lake Superior and Saskatchewan populations with some admixture (Fig. 2b) were added to assess whether *A. arenicola* had more likely emerged from Lake Superior *A. lyrata* and shared secondary contact with *A. lyrata* from Saskatchewan or vice versa. The better model was the one with *A. arenicola* having split from northwestern Lake Superior populations and secondary contact of the two species in northern Saskatchewan. This best model (Fig. 2b) suggested a split time between the two species of 6007 generations ago (95% CI: 5551–9772, Supplementary Data 4) and therefore clearly after the LGM assuming a generation time of 1 to 2 years^33^. This time point is also close to the 10 kyr ago when the western part of north shore Lake Superior became ice-free^34^.

The latter demographic model also revealed estimates of gene flow between species that were relatively low and comparable in both directions. An exception was exchange from Lake Superior *A. lyrata* (LSa) to *A. arenicola*, showing an approximately five times higher migration rate in the best fitting model, but bootstrap confidences were large (Supplementary Data 4). Furthermore, Patterson’s *D*-statistic revealed no consistent gene flow from *A. arenicola* back to *A. lyrata*, with 5 out of 6 *A. lyrata* plants from northwestern Lake Superior showing no significant evidence of introgression (Supplementary Fig. 14). Results on gene flow suggest that initial speciation progressed rapidly after the budding of the new species, with little late-acting gene flow.

### Demographic history of North American *Arabidopsis*

We addressed the demographic history of populations in the area of speciation, in particular the mating system. The TreeMix tree (Fig. 2a) suggested that postglacial recolonization of northwestern Lake Superior started from an area delimited by basal western populations in Wisconsin (WI1, WI2) and northeastern Iowa (IA2), which greatly overlaps with the Driftless Area that was not covered by ice during the LGM. All populations of *A. lyrata* from this area were previously found to be predominantly outcrossing^30^. Furthermore, the TreeMix tree suggested fairly direct colonization of northwestern Lake Superior, without too many intermediate nodes. This colonization event must have been accompanied by a shift in mating system to selfing as all *A. lyrata* populations of northwestern Lake Superior were predominantly selfing^30^.

Reconstruction of historic population size dynamics suggested a selfing reproductive mode also in *A. arenicola* across its range. Historic effective population sizes were estimated based on genome-wide heterozygosity and coalescent modeling (Fig. 2c, Supplementary Figs. 15–24). PSMC output was consistent with a history of large population sizes in populations from the Driftless Area, with a peak around 125 kyr ago (Supplementary Fig. 15), during the previous interglacial period^34^. Peaks in historic population sizes were found in a similar time range in central and northern populations of eastern *A. lyrata* (Supplementary Figs. 16, 17). However, low heterozygosity indicating reduced effective population sizes was found in previously described selfing populations of *A. lyrata*, around Lake Erie (Supplementary Fig. 18, all except PA4; OH1 in Supplementary Fig. 19), in Missouri (MO2 in Supplementary Fig. 21) and in southern North Carolina (NC1 in Supplementary Fig. 22). In line, low heterozygosity, presumably due to selfing, was found for populations of *A. lyrata* subsp. *lyrata* of northwestern Lake Superior (MI6 and ON11.1 in Supplementary Fig. 23) and all populations of *A. arenicola* (Supplementary Fig. 24).

Microsatellite analysis on a larger sample of plants of three *A. arenicola* populations supported that also the new species was predominantly to completely selfing (Supplementary Data 5). Two populations from near Churchill, Manitoba, had inbreeding coefficients, *F*_IS_ of 0.48 and 0.58, which translate into multilocus outcrossing rates <0.2 based on the relationship between *F*_IS_ and multilocus outcrossing rate established in extensive progeny array analysis in *A. lyrata*^30^. For a third population from western Quebec, *F*_IS_ could not be determined because of very low polymorphism at the 19 loci, but progeny array analysis confirmed a multilocus outcrossing rate of 0.0 (standard deviation: 0.0). Efficient selfing in nature was also suggested by the finding that these three populations of *A. arenicola* as well as *A. lyrata* of northwestern Lake Superior selfed autonomously when raised in the greenhouse.

**Fig. 3:**
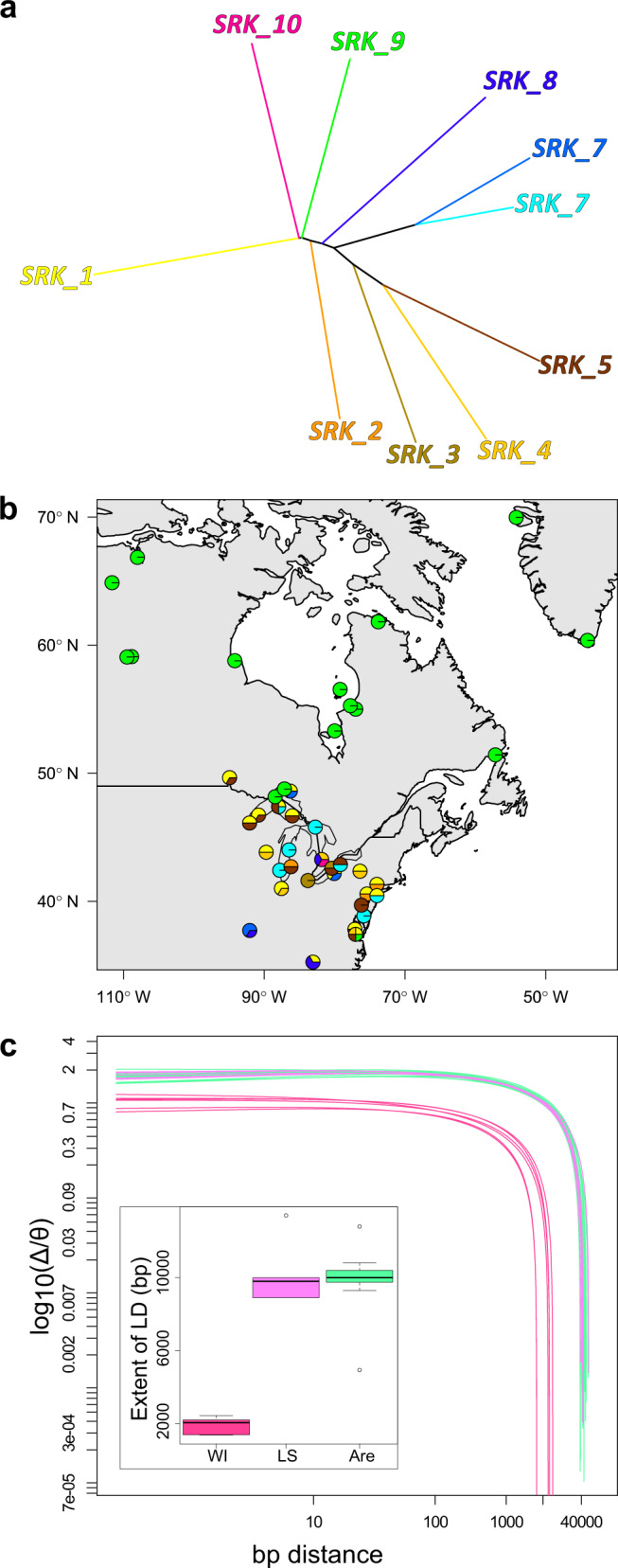
Haplotype diversity at the self-incompatibility (S-)locus, and genome-wide shift in linkage disequilibrium (LD). **a** Neighbor-joining tree of haplotypes of the S-locus gene *SRK*. **b** Map showing the distribution of the major haplotype groups of *SRK*. Haplotypes could only be reconstructed for some individuals (Supplementary Data 6). **c** Individual-based LD estimated by the correlation of zygosity (Δ) that was scaled by the level of genome-wide heterozygosity (θ). Colors depict *A. lyrata* individuals from Wisconsin (WI, *n* = 6) and north shore Lake Superior (LS, *n* = 6), and non-admixed *A. arenicola* (*n* = 15). Boxplots in the inset depict the extent of LD, estimated by the base pair (bp) distance over which the scaled log_10_-transformed Δ reached a low value of 0.1 (box plot elements: center line is the median, box limits are the upper and lower quartiles, whiskers the 1.5x interquartile ranges and points the outliers). Source data are provided as Source data files.

Evidence that the shift in mating system possibly involved the self-incompatibility (S-)locus in northwestern Lake Superior *A. lyrata* and was passed on to *A. arenicola* was provided by haplotype information. Genomic analysis of two genes flanking the S-locus (*ARK3*, *UBOX*) and one gene within the S-locus (*SRK*) revealed that *A. arenicola* had fixed alleles at all three loci and that they shared those alleles with *A. lyrata* populations of northwestern Lake Superior (MI6, ON11; Fig. 3a, b, Supplementary Fig. 25, Supplementary Data 6). In contrast, allelic diversity at these three genes was larger in *A. lyrata* in other areas of its distribution.

Probably primarily as a consequence of the range expansion and mating system shift, linkage disequilibrium (LD) was high. Individual-based LD estimated by the correlation of zygosity was high for Lake Superior *A. lyrata* and tended to be even higher in *A. arenicola* (Fig. 3c). The extent of LD was moreover similar among all non-admixed *A. arenicola*, independent of range expansion. We conclude that speciation of *A. arenicola* was preceded by a shift in mating system in parental *A. lyrata* of northwestern Lake Superior, which must have lost self-incompatibility during northern range expansion, and this shift led to the buildup of high linkage disequilibrium across the genome.

### Geographic pattern of mutational load

**Fig. 4:**
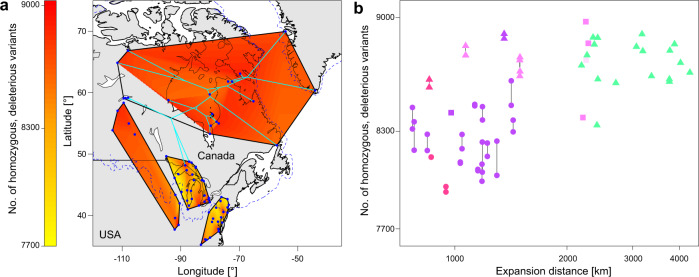
Mutational load. **a** Map of interpolated mutational load (number of homozygous, moderate- and high-impact variants) across the eastern and two western clades of *Arabidopsis lyrata* and *A. arenicola*. Interpolation was based on population means when information of two individuals were available (for most *A. lyrata* populations), or on one individual (all *A. arenicola* samples). Sampling sites are represented by blue dots. Also plotted in bright blue is the map-projected TreeMix tree, starting with the node from which the easterly mid-western populations of *A. lyrata* and all *A. arenicola* appeared. No interpolation was performed for Saskatchewan samples with evidence of admixture (gray area within minimum convex polygon hull of *A. arenicola*). **b** Mutational load plotted against the expansion distance of mid-western *A. lyrata* and *A. arenicola*. Symbols indicate the number of homozygous, deleterious variants for individuals, and lines connect samples from the same population (for *A. lyrata* only, except WI1.1, SK, AB). Colors indicate sample origin based on cluster assignment under K = 8 (Fig. 1b, on the right; Wisconsin in dark pink, Michigan with WI1.1 in velvet, un-admixed Saskatchewan in bright pink, *A. arenicola* in green, admixed individuals in pink). Selfing populations of *A. lyrata* and *A. arenicola* are depicted by tringles, outcrossing populations by circles; squares indicate that the mating system is unknown. While there is considerable variation in the number of homozygous, deleterious variants within and across populations of *A. lyrata*, related to expansion distance and mating system^31^, there is no further increase and relatively little variation in *A. arenicola*. Source data are provided as a Source data file.

Finally, we assessed the evolution of mutational load around the area of speciation and in *A. arenicola* along its expansion route. Based on TreeMix, population relatedness was plotted on the map to reveal expansion routes, within western *A. lyrata* and within *A. arenicola* (Fig. 4a). *A. lyrata* expanded its range from the Driftless Area to Lake Superior, and beyond to the Lower Michigan Peninsula, and over to Lake Huron and Lake Erie. Along this expansion route, mutational load gradually increased, and even more so in populations that shifted to a selfing mating system^31^ (Fig. 4b). Within *A. arenicola*, expansion happened along a main axis towards the northeast, with splits in western, northern and southeastern directions. During this long expansion, mutational load did not increase any further than levels achieved within northwestern Lake Superior *A. lyrata*, presumably because of purging or quasi-clonal selection associated with strong inbreeding. The latter is also supported by the low variation in mutational load within *A. arenicola*, compared to e.g., variation within some populations of *A. lyrata*.

## Discussion

Our phylogeographic analysis pointed to *Arabidopsis arenicola* being an example of peripheral parapatric speciation (Fig. 2a). During post-glacial northern colonization by the parental species *A. lyrata* of North America, *A. arenicola* emerged in the area of northwestern Lake Superior (Fig. 1e), at the periphery of the *A. lyrata* distribution. Subsequently, *A. arenicola* extended its range towards more northern areas with cooler conditions (Fig. 1d), probably tightly following the withdrawing ice sheet in northeastern direction. The two species may have initially remained in close parapatry, indicated by some evidence for introgression in a fraction of northwestern Lake Superior *A. lyrata* samples (Supplementary Fig. 14). Gene flow after budding may have led to the buildup of reproductive isolation, given the high to complete (depending on cross direction) intrinsic reproductive isolation between Lake Superior *A. lyrata* and southern *A. arenicola* found in a crossing experiment^29^. Later, the two species might have become separated by ecological succession of the vegetation to boreal forests between Lake Superior and the Hudson Bay. Furthermore, during later northwestern expansion of *A. arenicola*, the species came into secondary contact with *A. lyrata* in the area of Lake Athabasca, in northern Saskatchewan (Fig. 1d), where we located interspecific hybrids.

A shift to inbreeding had probably been an important pre-requisite for the speciation of *A. arenicola*. Theoretical models predict that the success of speciation with gene flow depends on the pre-existence or simple genetics of assortative mating, its strength and—associated with that—the buildup of linkage disequilibrium^35^. Self-compatibility and selfing in *A. lyrata* of northwestern Lake Superior and in *A. arenicola* fulfills these points. In *A. lyrata*, as in many hermaphroditic plants, close inbreeding and selfing is prevented by the self-incompatibility (S-)locus, with one gene responsible for recognition on the side of the pollen and one gene on the side of the pistil (*SRK*, Fig. 3a, b). But either by loss-of-function mutations at the S-locus or genetic change at modifier loci, selfing can evolve^36^. Self-compatibility segregates at low frequency in almost all outcrossing populations of *A. lyrata*^37^, suggesting that it evolved from standing genetic variation. Furthermore, selfing in *A. lyrata* was shown to be associated with about 2–2.5 times longer blocks of linkage across the genome, after accounting for differences in expansion history^38^. Linkage in northwestern Lake Superior *A. lyrata* and *A. arenicola* was equally high (Fig. 3c), about 5 times in extent as for an average Wisconsin *A. lyrata*. With a likely simple genetic basis of self-compatibility, standing genetic variation for that trait, and the buildup of linkage by range expansion and selfing, important elements for the initiation of parapatric speciation were latent.

Shifts in mating to inbreeding at range edges may not be uncommon. Range edge populations often have small size because habitat is rare and isolated^39^, or they have a history of small size due to past range expansion and serial bottlenecks^11^. Particularly expansions by serial bottlenecks may lead to evolutionary shifts to selfing. Under such conditions, genetic systems preventing close inbreeding and selfing such as self-incompatibility may break down in favor of reproductive assurance^16,40^. In plants, the shift from self-incompatibility to self-compatibility and selfing is a commonly observed evolutionary transition^36^. Also in some phyla of hermaphroditic animals, shifts between outcrossing and selfing are common^41^. Within parental *A. lyrata*, at least four geographically independent shifts to self-compatibility and selfing happened towards the endpoints of post-glacial range expansion and one in a rear edge region of distribution^30,42^. Similarly, a shift in mating system towards self-compatibility and self-fertilization was described in the context of postglacial range expansion in *Campanula americana*^43^. Many hermaphroditic species may show such a trend, but detection is hampered by a lack of range-wide investigations on mating system variation^44^.

Range expansions by serial bottlenecks were typical after Pleistocene glaciation cycles and may have often helped speciation. For several larger taxonomic groups, Pliocene climate change and Pleistocene glaciation cycles have been considered “pumps of speciation” causing phases of allopatry^11,13,14^: Species were split into separate populations associated with distinct refugia and became genetically differentiated over time, often promoting speciation. Here we show another mode of speciation triggered by Pleistocene glaciation. From one refugium a species expanded its range, which must have favored the evolution of selfing and heightened linkage. Two other favorable aspects of range expansion by climate change seem related with ecological opportunity. First, postglacial range expansion led species to areas with ample unoccupied habitat. This may be the reason that *A. arenicola* nowadays has a larger range than parental *A. lyrata*, a finding contrary to the common-held assumption of comparative biogeography that parapatric speciation leads to new species with smaller ranges compared to the parental species^45^. Second, if species track climate change closely, climate at the expansion front may remain similar. *Arabidopsis lyrata* and later *A. arenicola* seem to have followed the withdrawing ice sheet tightly. Therefore, divergent selection by warming towards the south must have occurred mostly in the back of the wavefront, at least for northern range expansion^46^, producing a demographic burden of climate adaptation behind but not at the wavefront. A similar situation was recently described for turtles during the Eocene−Oligocene cooling. While a fraction of turtle species went extinct in drying inland parts of continents, many new species emerged in the newly appearing continental margin habitat^9^. For the latter, two favorable aspects were emphasized for speciation: range expansion into newly exposed, unoccupied habitat and conditions that were similar to those ancestors had experienced.

Finally, the mode of speciation we outlined here is associated with little increase in mutational load. In a past study on range expansion in *A. lyrata*, an increase in drift load along the expansion routes in outcrossing populations was documented, and an additional increase in load with a shift to selfing^31^. Estimates of load based on coding DNA sequences were correlated with expressed load estimated by heterosis, indicating that load indeed had fitness consequences. However, as the range expansion towards northwestern Lake Superior was relatively short, the load that *A. lyrata* populations had accumulated by then was low compared to other regions where selfing evolved later along the expansion route^31^. The generally low load at the onset of speciation likely helped the speciation process. Furthermore, the populations we currently find at the northwestern range edge of *A. lyrata* and populations of *A. arenicola* have similar drift load (Fig. 4), indicating that over the 3000-km-long expansion of the new species, load did not systematically increase. The reason must be that selfing can lead to the purging of mutation load^47^.

In conclusion, we outline that peripheral parapatric speciation is an important scenario of speciation under climate perturbations, or any major environmental transition that leads to the opportunity of range expansion (Supplementary Fig. 26). At the wavefront, ample empty habitat may become available with conditions that remain constant if movement is synchronized with climate/environmental change. If expansion is rapid, serial bottlenecks may occur that favor the (evolutionary) transition towards more inbreeding and selfing. In turn, inbreeding or selfing are a form of assortative mating leading to the buildup of linkage favoring the initiation of the speciation process. In the back of the wave front of expansion, climate and other gradients establish and can cause divergent selection that add to promote speciation (or lead to extinction). This hypothesis should be tested further in species living e.g., in the arctic or in habitat that became available vastly and rapidly.

## Methods

### Plant material and sampling strategy

We collected plant material from 54 populations of *A. lyrata* subsp. *lyrata* and 1 population of *A. arenicola* during the reproductive seasons of 2007, 2011, 2013, and 2014, and we selected two individuals per population for whole-genome resequencing. Additional samples were added from herbarium vouchers of the Department of Agriculture Ottawa, DAO, particularly for *A. lyrata* subsp. *lyrata* from the Canadian Prairie Provinces, and for *A. arenicola* from across the range. We also included samples from closely related species as outgroup for phylogenetic analysis of the plastid genome (Supplementary Methods 1). We downloaded the raw reads from SRA at NCBI/GenBank for taxa that had been used in a genus-wide sequencing project^28^. Details on samples are given in Supplementary Data 1.

### DNA extraction and library preparation

DNA was extracted using the DNeasy Plant Mini Kit (Qiagen) following the manufacturer’s instructions and quantified with the Qubit 2.0 fluorometer using the dsDNA HS Assay Kit (ThermoFisher Scientific). Paired-end whole-genome sequencing libraries with insert size 300–500 bp were prepared at the Genomics Facility of the D-BSSE in Basel, Switzerland, using the KAPA HyperPrep kit (Kapa Biosystems). Libraries were then sequenced with read length 151 bp on an Illumina HiSeq2500 machine (Illumina Inc.). For samples from silica-dried leaf material, every sequencing library was sequenced twice, in two independent runs, to mitigate potential sequencing bias. All samples from herbarium vouchers were sequenced on a single lane.

### SNP calling

Trimmed paired-end reads were mapped against the reference with bwa-mem of bwa v. 0.7.13^48^ with default settings. The *A. lyrata* reference genome^49^ was used as a reference with minor modifications: The complete mitochondrial genome from *A. thaliana* was added (ENA/GenBank accession NC_001284), as well as a plastid genome from *A. lyrata* (ENA/GenBank accession LN877383) with the second copy of the Inverted Repeat removed, thus truncating it to a length of 128,646 bp. In addition, positions 8,746,475–8,835,273 and 9,128,838–9,212,301 on scaffold_2 of the nuclear genome were masked because of their high similarity to the chloroplast genome. Following mapping, alignments were sorted and subsequently merged by individual with SAMtools v. 1.3.1^50^. After filtering for technical duplicates, for regions with excessive coverage, and indel realignment (described in Supplementary Methods 2), mean coverage of the newly sequenced samples was between 5 and 15 for the nuclear genome, between 476 and 6486 for the plastid, and between 24 and 599 for the mitochondrion (Supplementary Fig. 27). The four allopolyploid *A. kamchatica* were excluded from analysis of the nuclear genome, resulting in 155 samples for the complete nuclear dataset. Finally, variants (both SNPs and InDels) were called with GATK UnifiedGenotyper v. 3.7^51^ for every sample and genome separately. For the nuclear genome, we set the ploidy to 2, for mitochondrial genome and plastid genome to 1. Only sites with minimum base quality 28 were called (further filtering described in Supplementary Methods 2). Variants were finally annotated with SnpEff^52^.

### Analysis of population structure

Population structure of North American *A. lyrata* and *A. arenicola* was analyzed with ADMIXTURE v. 1.3.0^53^. We restricted sites to biallelic SNPs with minor allele count >3 to exclude the less informative autapomorphies, and to synonymous positions across the 136 samples. In addition, we used VCFtools^54^*—thin* to obtain a dataset for which the minimum distance between two loci was 20 kb so as to avoid sites in linkage. This resulted in a set of 7781 SNPs. We ran ADMIXTURE for K 1–10 with 200 bootstrap replicates (by the block bootstrap method) and used cross validation to determine the best number of clusters (results in Supplementary Fig. 1).

### Nuclear genome trees

We constructed a phylogenetic tree based on the nuclear genome using a similar filtering scheme as for analysis of population structure; due to the inclusion of outgroup samples 8187 SNPs remained. We used GATK FastaAlternateReferenceMaker to construct fasta sequences containing only the selected SNPs and inserting IUPAC ambiguity codes for heterozygous sites. A maximum likelihood phylogenetic tree was reconstructed using RAxML v. 8.2.8^55^ excluding samples from North America showing more than 5% admixture at K = 5. A rapid bootstrap analysis with 1000 bootstrap replicates and subsequent maximum likelihood search was conducted (RAxML command line option *-f a*). GTRGAMMA was used as the substitution model, and *A. thaliana* was set as outgroup. Estimation of divergence time and phylogenetic reconstruction based on organellar genotyping is described in Supplementary Methods 3 and 4.

As admixture can strongly affect the placement of individuals in phylogenetic trees, we also used TreeMix v. 1.13^56^ for phylogenetic reconstruction. This method reconstructs phylogenies while explicitly accounting for gene flow. Here, sampling was restricted to North American *A. lyrata* and *A. arenicola*, with one European *A. lyrata* subsp. *petraea* from Germany as outgroup. The analysis was restricted to biallelic sites like above; however, since TreeMix accounts for linkage in the genome by grouping SNPs, we did not subsample sites based on distance; rather, we used a block size (-k) of 250 SNPs. The software was run with a number of migration events from none to five (number of admixture zones detected in the best supported ADMIXTURE run). Following the manual, we analyzed the plots of residuals to determine the number of migration events best supported by the data.

### Modeling species split and early demography using fastsimcoal

To reconstruct the precise scenario of *A. arenicola* splitting from North American *A. lyrata*, we implemented hierarchical modeling in fastsimcoal2 v. 2.6^57^ (detailed description in Supplementary Methods 5). Scenarios included up to six super-populations, each comprising data from one fully re-sequenced individual of three nearby populations (Supplementary Data 2), for which unfolded site frequency spectra (SFS) were calculated following Marchi et al.^58^. Expected SFS for each model and parameter combination were approximated with 100,000 coalescence simulations and 100 expectation conditional maximization (ECM) cycles with a stopping criterion of 0.001 for each run. We then obtained the likelihood and parameter estimates for each model from the run with the highest likelihood among 100 optimization steps. Concurrent models were compared using the Akaike information criterion (AIC).

Scenarios were compared in four sequential, hierarchical steps. A first set of scenarios established the topology of the three main western super-populations of Missouri (MO), Saskatchewan (SK), and Wisconsin (WI). A second set explored the location of un-admixed *A. arenicola* relative to MO, SK, and WI. The third set compared the timing of the split of the younger populations including *A. arenicola*. Finally, the fourth set of scenarios was on secondary contact and gene flow and included *A. lyrata* populations with a signature of admixture in cluster analysis (K = 5), namely Saskatchewan (SKa) and north shore Lake Superior (LSa). The four steps are described and illustrated in more detail in Supplementary Methods 5 and Supplementary Fig. 13. Evidence for gene flow was tested by Patterson’s *D* statistic (detailed description in Supplementary Methods 6).

### Analysis of past population size, mating system, LD, and mutational load

Historical population size dynamics was revealed for each population of *A. lyrata* and samples of *A. arenicola* by pairwise sequentially Markovian coalescent models^59^. The software PSMC makes use of the local density of heterozygous sites and their changes across the genome to infer the distribution of time since the most recent common ancestor and the history of change in population size over time. We ran PSMC using 100 bootstrap replicates and the following parameters: -N25 -t5 -r5 -p 4 + 25 × 2 + 4 + 6 (detailed description in Supplementary Methods 7).

The mating system of three *A. arenicola* populations was assessed following Griffin and Willi^30^ based on microsatellite genotyping and the calculation of the population inbreeding coefficient, or by progeny array (Supplementary Methods 8). Furthermore, allelic diversity at the S-locus was investigated by obtaining haplotypes of the two flanking genes (*ARK3, UBOX*) and a gene that is part of the S-locus (*SRK*), following the procedure of Genete et al.^60^ (Supplementary Methods 9). Because *ARK3* and *SRK* have highly polymorphic regions, we focused on the ~1 kb sequence coding for the extracellular region.

Linkage disequilibrium for individual sequences was estimated by the correlation of zygosity, the strength of correlation between loci within individual genomes^61^. The correlation of zygosity (Δ) was calculated across genic regions following Lucek and Willi^38^ and compared among non-admixed *A. arenicola* and *A. lyrata* plants from Wisconsin (WI) and northwestern Lake Superior (LS) (Supplementary Methods 10). For each individual, we also calculated the distance in bp over which the log_10_-transformed Δ reached a value of 0.1.

Mutational load was estimated by analyzing variant annotation obtained from SnpEff^52^. For all individual-sequenced samples, reads were aligned to the *A. thaliana* reference genome, and the same filtering steps as outlined above were applied. SNP calling was performed for all samples together, which were then filtered for genotyping quality and coverage; no missing data was allowed. Annotation by SnpEff was done without considering up- and downstream annotations. Homozygous moderate- and high-impact variants were summed up for each sample; moderate variants included missense changes, and high-impact variants included changes in start and stop codons. Both types of variants were added, and the mean of samples within *A. lyrata* populations was taken, and values were plotted for western samples of *A. lyrata* and for *A. arenicola* by interpolation (Fig. 4a). Furthermore, log-transformed expansion distance was related with individual mutational load (Fig. 4b). The topology of the TreeMix tree was used to project past range expansion of the new species on the map, starting from the node splitting the two near-western clades of *A. lyrata*, with one including *A. arenicola*. Starting points for colonization after the LGM within *A. lyrata* had been revealed similarly^31^. Geodesic distances were calculated from the endpoints of the western part of the TreeMix tree back to the origin of western post-LGM expansion.

Figures, when not an output of software listed, were produced with R v. 4.1.2^62^. R packages downloaded in spring 2022 and used for creating plots were: akima, ape^63^, fields^64^, gdalUtils, grDevices, lmomco^65^, mapdata, mapplots, maps, maptools, phytools^66^, plotrix^67^, PBSmapping, rgdal, rgeos, and sp^68,69^. Data files for mapping came from: http://www.naturalearthdata.com/downloads/10m-physical-vectors/10m-rivers-lake-centerlines/—for lakes, http://gadm.org/—for country, state, and province boundaries, http://geoscan.nrcan.gc.ca/starweb/geoscan/servlet.starweb?path=geoscan/downloade.web&search1=R=214399—for ice sheet extent.

### Reporting summary

Further information on research design is available in the Nature Portfolio Reporting Summary linked to this article.

## Supplementary information

Supplementary Information

Description of Additional Supplementary Files

Supplementary Data 1-6

Reporting Summary

## Data Availability

Raw sequences of newly sequenced individual plants are available under the ENA/GenBank projects PRJEB30473 and PRJEB23202 (see Supplementary Data 1). Sequences of further plants were accessed from ENA/GenBank (see Supplementary Data 1). The authors declare that all other data supporting the findings of this study are available within the article and its supplementary information files. Source data are provided with this paper.

## Source data

Source Data
