# Supplementary material for: Recent speciation associated with range expansion and a shift to self-fertilization in North American Arabidopsis
Source: Nat Commun. 2022 Dec 8;13:7564. doi: 10.1038/s41467-022-35368-1 (PMC9732334; doi:10.1038/s41467-022-35368-1)
Supplement: Supplementary file 1 — Supplementary Information [file 41467_2022_35368_MOESM1_ESM.pdf]

Supplementary Information

Recent speciation associated with range expansion and a shift to self-fertilization in North American *Arabidopsis*

Yvonne Willi*, Kay Lucek, Olivier Bachmann, Nora Walden

*Corresponding author, email address: yvonne.willi@unibas.ch

Contents

- Supplementary Methods
- Supplementary Note
- Supplementary Figures 1 - 27
- Supplementary References

Supplementary Methods

Contents

1 *Additional samples* 2

2 *Filtering of DNA sequence data* 2

3 *Divergence time estimation based on nuclear SNPs* 3

4 *Organellar genotyping and phylogenetic reconstruction* 4

5 *Modeling species split with fastsimcoal* 5

6 *Analysis of admixture between* A. lyrata *and* A. arenicola *by ABBA-BABA testing* 7

7 *Analysis of past population size with PSMC* 8

8 *Analysis of mating system of* Arabidopsis arenicola *populations* 8

9 *Reconstruction of allelic diversity at the S-locus* 9

10 *Linkage disequilibrium* 10

1 Additional samples

The samples included from Novikova et al.[1] were: three individuals from *A. arenicola*, seven individuals from European sister species *A. lyrata* subsp. *petraea*, four individuals from *A. kamchatica*, an allotetraploid hybrid with *A. lyrata* as the paternal species and distributed in Beringia, one individual of *A. cebennensis*, a small-range endemic from France, and finally three individuals from *A. petraea* (one from subspecies *umbrosa*, two from subspecies *septentrionalis*), a close relative of *A. lyrata* from Russia. Details on samples are given in Supplementary Data 1.

2 Filtering of DNA sequence data

Trimming for quality and adapter sequences was performed with trimmomatic v. 0.36[2] with the following settings: phred-based quality 28 in a sliding window of 8 bp, leading and trailing quality 20, minimum length 50 bp. Reads from the same individual sequenced in two independent runs were processed separately. Only paired reads were used for downstream analyses.

After mapping and merging sequences by individual plant, PCR duplicates and optical duplicates were removed with MarkDuplicates and sorted by coordinate with SortSam from picard v. 2.8.0 (https://broadinstitute.github.io/picard/). Indel realignment was performed in GATK v. 3.7[3] with downsampling disabled, and secondary alignments were excluded from the mapping with SAMtools view (-F 256). Next, regions with excessive coverage, such as can be the result of repetitive regions, were also excluded. To determine intervals with intermediate coverage, we calculated coverage density from pileup files (excluding zero coverage regions): We used GATK to generate a pileup file including the eight main scaffolds, the plastid and mitochondrial genomes, and then calculated coverage density for the three genomes separately. For each sample we then selected genomic regions with intermediate coverage using GATK

CallableLoci with minimum depth 3 and maximum depth corresponding to the 0.98 percentile of the coverage distribution, considering only reads of mapping quality of at least 20.

After variant (both SNPs and InDels) calling with GATK UnifiedGenotyper for each sample and genome separately, strict hard filters for mapping quality and strand bias were applied. We used GATK VariantFiltration to exclude sites using the following criteria: QualByDepth (QD) < 2.0, MapQual (MQ) < 40.0, FisherStrand (FS) > 60.0, StrandOddsRatio (SOR) > 3.0, MapQualRankSum (MQRankSum) < -12.5, ReadPosRankSum < -0.8. Additionally, previously detected repetitive parts of the nuclear genome[4] were excluded using bedtools[5], and biallelic SNPs were selected for downstream analysis. For phylogenetic analyses of the nuclear genome and analysis of the direction of gene flow, we also called nuclear variants for all 155 diploid samples (excluding tetraploid *A. kamchatica*) and for all samples from North America including the respective outgroup samples simultaneously and filtered the multi-sample vcf using the same parameters. We restricted this variant calling to sites of intermediate coverage in all samples and with no missing data allowed.

3 Divergence time estimation based on nuclear SNPs

We used the SNAPP plugin[6] in BEAST v. 2.6.2[7] to estimate divergence times based on nuclear SNPs. We excluded all samples showing > 5% admixture at K = 5 and added three outgroup samples for calibration (*A. thaliana*, *A. halleri* and European *A. lyrata* subsp. *petraea*). To both avoid linked sites and achieve a computationally feasible dataset, we selected synonymous SNPs with a minimum distance of 100 kb. The input file was generated using the snapp_prep.rb script (https://github.com/mmatschiner/snapp_prep), with samples grouped on the population level and using secondary calibration from the plastid divergence time analysis. Specifically, three calibration points were set as follows: The divergence of *A. halleri* was constrained using a lognormal distribution with a mean (in real space) of 0.7166631 and sd (log) of 0.1554624,

divergence of *A. lyrata* subsp. *petraea* with mean 0.4594802 and sd 0.1738735, and the crown age of North American *A. lyrata* with mean 0.2941328 and sd 0.162305. Additionally, we set theta to be estimated. A chain length of 100'000 generations was used, sampling every 100 generations, and discarding the first 10% as burn-in. Eight independent analyses were run and combined using LogCombiner and TreeAnnotator. The results were visualized in FigTree v. 1.4.4.

4 Organellar genotyping and phylogenetic reconstruction

Plastid genome sequences were retrieved using GATKs FastaAlternateReferenceMaker. The complete sequence was then aligned using MAFFT v. 7.164[8] with automatically determined settings (FFT-NS-2 algorithm). To exclude poorly aligned regions and indels, we used Gblocks v. 0.91[9] with minimum block size 5 and number of sequences for flanked or conserved position set to the number of samples, resulting in a total alignment length of 127'459 bp. We used RAxML v. 8.2.8[10] for phylogenetic reconstruction of the plastid genome with rapid bootstrap analysis using 1000 bootstrap replicates followed by rapid maximum likelihood search for the best scoring tree, applying the GTRGAMMA substitution model. Plastid genomes from North American *A. lyrata* showed very little intrapopulation variation; all plastomes were identical or closely related to that of the other individual from the same population with few substitutions. For tree reconstruction, we therefore only included the first individual of every population on the list, and other identical sequences were discarded as well using the reduced alignment from RAxML.

For the mitochondrial genome, which is structurally highly variable in plants and contains many noncoding and repetitive sequences, we only evaluated SNPs in coding sequences (CDS) annotated on the *A. thaliana* reference genome. Alignment was constructed by concatenating the sequences obtained using GATK's FastaAlternateReferenceMaker. The

final mitochondrial alignment was 36’018 bp in length and consisted of 216 variant sites. A ML phylogenetic tree was reconstructed in RAxML using the same parameters as for the plastid genome. Results are presented in Supplementary Fig. 6.

Divergence time estimation based on the plastid genome data was carried out in BEAST v. 1.8.3[11]. Calibration points for secondary calibration were extracted from a previous study estimating divergence times in *Arabidopsis*[1]. We fit lognormal distributions at four nodes of their trees to obtain the respective means and standard deviations for calibration in *Arabidopsis* with *fitdist* from R package fitdistrplus[12]. The root height (corresponding to the split between *A. thaliana* and the rest of the genus) was calibrated with meanlog 1.7381 and sdlog 0.132, the crown age of the x=8 clade with meanlog -0.0155 and sdlog 0.2005, the split between samples lyrON4 (an individual from the same population as Alyratalyrata1[1]) and Aarenicola1 with meanlog -1.5067 and sdlog 0.2399, and finally the crown age of *A. lyrata* (split between main lineages, here lyrON4 and Acarpatica3) with meanlog -0.5107, sdlog 0.1882. We used a lognormal relaxed clock with estimated rates, the GTR+G+I substitution model, and the Birth-Death Incomplete Sampling tree model[13].

5 *Modeling species split with fastsimcoal*

To reconstruct the most likely scenario of *A. arenicola* splitting from North American *A. lyrata*, we implemented a hierarchical model approach in fastsimcoal2 v. 2.6[14] and compared the fit of different simulated models of population splitting to observed site frequency spectra (SFS). Unfolded SFS were generated with easySFS (https://github.com/isaacovercast/easySFS) based on unascertained polymorphic and likely neutral sites that were filtered from our overall dataset[15]: We removed CpG sites and sites found in CpG islands (defined as regions >100 bp with a moving average CpG frequency > 0.06[16]), and then filtered for A<->T and G<->C sites

that were polymorphic in our selected samples. The dataset mapped to the *A. thaliana* reference was used to allow for polarization.

Scenarios included up to six broader “super-populations”, each comprising data from one fully re-sequenced individual of three nearby populations or sample locations, with similar history based on the TreeMix tree (Supplementary Data 2). The main reason for pooling was to lower overwhelming drift effects since population splitting, particularly in the case of selfing populations. On a practical side, we typically had only 1 individual sampled at a site for *A. arenicola*, which required pooling. Pooling can create a Wahlund effect, with faster coalescence for homologous alleles within a population (or selfing lineage) relative to alleles of different populations. Therefore, we introduced population inbreeding coefficients in our modelling as nuisance parameters[15], however only for the groups of (presumably) selfing populations of *A. lyrata* and for *A. arenicola*.

We approximated the expected SFS for each model and parameter combination with 100’000 coalescence simulations and 100 expectation conditional maximization (ECM) cycles with a stopping criterion of 0.001 for each run. We then obtained the likelihood and parameter estimates for each model from the run with the highest likelihood among 100 optimization steps. For each hierarchical step (Supplementary Fig. 13), we compared concurrent models using the Akaike information criterion (AIC).

For all models, we provided the same initial parameter ranges for effective population size (N_e), which we approximated from empirical estimates: We calculated θ for each single genome with mlRho v. 2.9[17,18] (see section 1.10) and then calculated N_e based on the equation: $\theta = 4N_e\mu$[19]. We assumed a mutation rate μ of 3.55e-9, the latter from *A. thaliana*, being the only available empirical estimate in this genus[20]. Because *A. arenicola* as well as several populations of *A. lyrata* are selfing[21], we further allowed for inbreeding in populations of *A. arenicola* (Aare), north shore Lake Superior (LSa) and Saskatchewan (SK) in all of our models.

In a first step we established the demographic relationship among the three unadmixed *A. lyrata* super-populations from Missouri (MO), Saskatchewan (SK) and Wisconsin (WI) (Supplementary Fig. 13; Supplementary Data 3), where one population is old, and the two others emerged since the last glaciation period. Given the patterns observed in our TreeMix analyses (Fig. 2a), we ran the models allowing either past gene flow or not. In a second step, we aimed to place the split between *A. arenicola* and *A. lyrata*, comparing all four concurrent models. Next, we tested if the split between *A. arenicola* and *A. lyrata* was more likely to have occurred recently, *i.e.*, since the last glaciation period, or prior to the last glaciation. Finally, we included individuals of two *A. lyrata* population groups that had shown admixture with *A. arenicola* in our other genomic analyses, of north shore Lake Superior (LSa) and Saskatchewan (SKa) (Fig. 1b, d, e). We modeled two concurrent scenarios that included a recent split of *A. arenicola* with past gene flow from one of the admixed *A. lyrata* populations and secondary contact with current gene flow with the other. For the finally selected model, we estimated the confidence intervals of the parameters from 100 non-parametric block-bootstrap replicates of the observed SFS. We performed 50 parameter optimizations with each bootstrap replicate, starting from the best parameters inferred from the observed data, and the parameter estimates from the run with highest likelihood for each bootstrap sample was used to compute 95% confidence intervals using empirical percentiles.

6 *Analysis of admixture between* A. lyrata *and* A. arenicola *by ABBA-BABA testing*

The presence of gene flow was additionally confirmed using Patterson's D statistic[22] on 8'199 SNPs selected following the same criteria as for ADMIXTURE and SNAPP, with *A. thaliana* acting as the outgroup. We split the North American *A. lyrata* and *A. arenicola* samples into admixed individuals based on ADMIXTURE results (admixture $\geq$ 5% at K = 5) as well as groups of non-admixed individuals (admixture < 5% at K = 5) according to their cluster

assignments. We tested if there was significant admixture between each admixed individual and its primary and secondary contributing cluster using all permutations of individuals from these two clusters. The 'CalcD' function from R package evobiR[23] was used to calculate Patterson's D. Significance was tested using bootstrapping with 1000 replicates, and admixture was considered confirmed when the mean P-value among permutations was below 0.05.

7 *Analysis of past population size with PSMC*

Historical population size can be estimated from individual sequencing data when coverage is reasonably high, a high-quality reference genome is available and generation time as well as mutation rate are known. We used PSMC[24] (the Pairwise Sequentially Markovian Coalescent model) to analyze past population size from selected individuals across the species range. Diploid consensus sequences were generated from bam files using vcf2fq from the vcfutils.pl collection in bcftools[25], with a minimum depth of 8 and a maximum depth corresponding to the 0.98 percentile of the coverage distribution. The PSMC input files were generated using the fq2psmcfa script from the psmc package with a minimum quality of 20. We ran PSMC using 100 bootstrap replicates and the following parameters: -N25 -t5 -r5 -p 4+25x2+4+6. PSMC output is scaled with generation time and mutation rate. We assumed a generation time of 2 years[26]. *Arabidopsis lyrata*, like most *Arabidopsis* species except *A. thaliana*, is a perennial, and exact generation time is unknown. We furthermore used the mutation rate from *A. thaliana*, which was estimated to be 7.1 x 10^{-9} using mutation accumulation lines[20].

8 *Analysis of mating system of* Arabidopsis arenicola *populations*

Green tissue of 30 to 33 plants of three populations of *A. arenicola* were sampled in 2013 and 2017, two in Churchill, Manitoba, and one in Blanc-Sablon, Western Quebec. DNA was extracted and genotyped at 20 microsatellite loci, but Lyr417 was excluded from later

analysis[21] (Supplementary Data 5). The mating system of the two *A. arenicola* populations from Churchill was deduced from the population inbreeding coefficient. For the population of Blanc-Sablon, only one locus was polymorphic, with the second allele occurring once in a heterozygous individual, such that the inbreeding coefficient could not be calculated. For that population, the mating system was assessed by progeny array, by raising 5-6 offspring per field-collected plant from seeds in the greenhouse. Offspring tissue was collected, DNA extracted and genotyped (Supplementary Data 5), and finally analyzed together with the parental genotype information with the program MLTR[27].

9 Reconstruction of allelic diversity at the S-locus

To capture the genetic diversity at the S-locus, we reconstructed the haplotypes of the two flanking genes (*ARK3, UBOX*) and a gene that is part of the S-locus (*SRK*). We first applied kmer filtering to extract reads similar to reference sequences for each gene obtained from GenBank including known paralogs with kmerRefFilter.py[28]. For each individual and gene, we then performed a *de novo* assembly with SPAdes v. 3.10.1[29] with the parameters: -k 21, 41, 81 and -careful. We aligned the obtained contigs back against the reference sequences with YASS v. 1.14[30], which allows alignment even for sequences with distant similarity. We retained only highly matching contigs, excluding all paralogs. For each gene, we performed an alignment with MAFFT[8] and generated a neighbor-joining tree. Because *ARK3* and *SRK* have highly polymorphic introns, we focused on the ~1kb sequence coding for the extracellular region. This approach allowed us to reconstruct 87 haplotypes for 87 individuals for *ARK3*, 124 haplotypes for 108 individuals for *SRK*, and 109 haplotypes for 109 individuals for *UBOX* (see Supplementary Data 6 for details). Assembly failed for other individuals. This may be because the sequencing depth of this genomic region was not high enough or because the actual sequence is too divergent to be recovered by kmer filtering.

10 Linkage disequilibrium

We calculated the correlation of zygosity (Δ) across all genic sites with mlRho v. 2.9[17] for all non-admixed *A. arenicola* individuals and compared those with values of individuals from Wisconsin (WI) and northwestern Lake Superior (LS). The correlation of zygosity measures the strength of correlation between loci across individual genomes and provides an individual-based estimate for LD[31]. However, rather than resulting in separate pairwise estimates, mlRho measures Δ across all loci for each individual. We included only sites with a minimal genotype quality of 28 and a sequence depth between five times and twice the average genome-wide depth for each individual[31]. We ran mlRho in steps of 1 bp along the first 5'000 bps and in steps of 100 bps for a distance of 5-50k bps. We scaled Δ by the level of heterozygosity (θ) estimated for each genome by mlRho as $\Delta/\theta \cong r^2$ [31]. We estimated for each individual the distance in bps where the $\log_{10}$-transformed Δ reached a value of 0.1[18].

Supplementary Note

Contents

Results (additional information) 2

1 *Results on population structure* 2

2 *Phylogeographic history of the* Arabidopsis lyrata *complex based on nuclear data* 2

3 *Phylogeographic history of the* Arabidopsis lyrata *complex based on organellar data* 3

4 *The phylogeographic history of North American* Arabidopsis lyrata *with TreeMix* 3

Results (additional information)

1 *Results on population structure*

Assignment analysis by ADMIXTURE indicated that North American *A. lyrata* subsp. *lyrata* and *A. arenicola* split best in 5 clusters (K = 5) (Supplementary Fig. 1). They were: *A. arenicola* from most of its known distribution; *A. lyrata* subsp. *lyrata* from Missouri, Saskatchewan and Alberta; *A. lyrata* subsp. *lyrata* mostly of Lake Michigan and nearby areas; *A. lyrata* subsp. *lyrata* from Virginia to New York State; and finally *A. lyrata* subsp. *lyrata* from North Carolina and Tennessee (Fig. 1b, c). Populations with signatures of admixture between *A. arenicola* and *A. lyrata* subsp. *lyrata* were found in northern Saskatchewan and on the north shore of Lake Superior. Similar clustering was obtained using the next best number of clusters (K = 8), with the three additional clusters consisting of the populations showing high levels of admixture at K = 5 (Fig. 1b, c). These additional clusters were: the admixed northern Saskatchewan and Lake Superior populations; populations of Wisconsin; and populations on Lake Erie.

2 *Phylogeographic history of the* Arabidopsis lyrata *complex based on nuclear data*

The maximum likelihood tree based on nuclear single nucleotide polymorphisms (SNPs) and excluding admixed individuals of *A. arenicola* and *A. lyrata* subsp. *lyrata* grouped the circumpolar *A. lyrata* complex into five clades largely congruent with geography: a northern European clade and a central European clade being sister to the three clades of western Russia, eastern Russia, and North America (Supplementary Fig. 2). Within North America, the split between eastern and western populations received good bootstrap support. In the eastern clade, the populations of North Carolina and eastern Tennessee emerged last and in sequence from north to south, indicating colonization after the end of the last glacial maximum as earlier emerging populations were located in areas covered by ice during the last glacial maximum.

The western populations were further split into three clades corresponding to the Missouri-Saskatchewan, the Lake Michigan, and the *A. arenicola* cluster, but branches were not supported well in bootstrap analysis. Divergence time estimates had considerable confidence intervals. The one between European *A. lyrata* subsp. *petraea* and North American *A. lyrata* subsp. *lyrata* with *A. arenicola* was around 349 kya (Supplementary Fig. 3).

3 *Phylogeographic history of the* Arabidopsis lyrata *complex based on organellar data*

A phylogeny on complete plastomes of *Arabidopsis* (sub-)species of the northern hemisphere pointed to a divergence time of 460 kya of North American taxa from European ones (Supplementary Figs. 4, 5). Apart, phylogenetic reconstruction based on plastomes and mitochondrial genes (Supplementary Fig. 6) revealed little resolution, pointing to a general lack of lineage sorting.

4 *The phylogeographic history of North American* Arabidopsis lyrata *with TreeMix*

Complementary insights about recent splits within North America were gained from a tree based on population allele frequencies of the nuclear genome that accounted for gene flow and also included populations with a signature of admixture. Similar to admixture analysis, the number of migration events for each analysis was a fixed input parameter. From m = 4 onward, residuals were uniformly distributed, indicating that four migration events were a good fit (Supplementary Figs. 7-12). The tree clearly showed that *A. arenicola* evolved from common ancestors of *A. lyrata* subsp. *lyrata* populations of the north shore of Lake Superior, Isle Royal (MI6) and Terrace Bay (ON11) (Fig. 2a). Migration events detected using TreeMix at m = 4 were largely consistent with gene flow detected in admixture analyses. One migration event was detected between *A. lyrata* lyrSK4 from Saskatchewan to a group of two *A. arenicola* and

one *A. lyrata* subsp. *lyrata* of the same area. Noteworthy is that Lake Erie populations emerged after the east-west split and took a basal position in the east, which we think is because admixture since colonization – the area was under ice during last glacial maximum (LGM) – probably involved high contributions of both eastern and western cluster.

Supplementary Figures

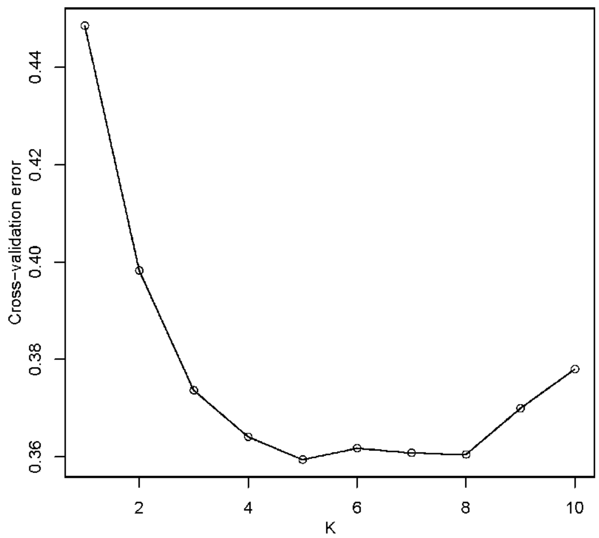

Supplementary Figure 1. ADMIXTURE analysis cross-validation error. Line plot depicting the cross-validation error of ADMIXTURE analysis for K = 1-10. Low cross-validation error indicates a good representation of data in the model; in this case, K = 5 and K = 8 represent the best choices for K-values and are thus discussed further. Source data are provided as a Source Data file.

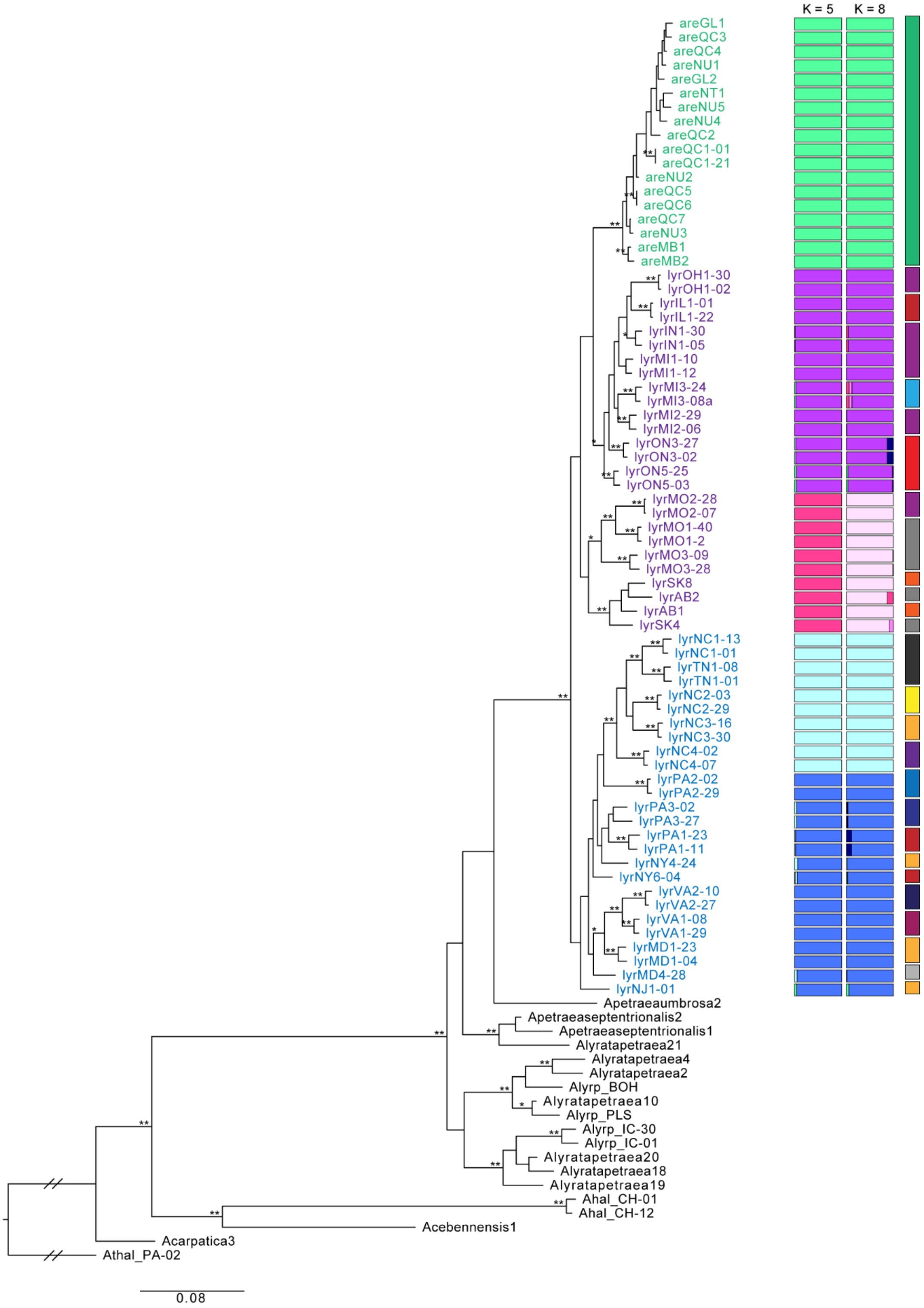

Supplementary Figure 2. Nuclear maximum likelihood phylogeny. Phylogenetic reconstruction was conducted with RAxML from synonymous, biallelic sites with a minimum distance of 10 kb. Only individuals with < 5% admixture at K = 5 were included. ADMIXTURE results and plastid haplotypes are shown on the right. Individual names are colored according to their origin for North American *Arabidopsis lyrata* (abbreviated with lyr; eastern cluster blue, western cluster purple) and *A. arenicola* (are; green). Their closest relatives were 4 samples from Russia (including *A. petraea* subsp. *umbrosa* from eastern Yakutia), supporting colonization of North America via Beringia. Bootstrap support from 1000 bootstrap replicates: ** 100; * 95-99. Source data are provided as a Source Data file.

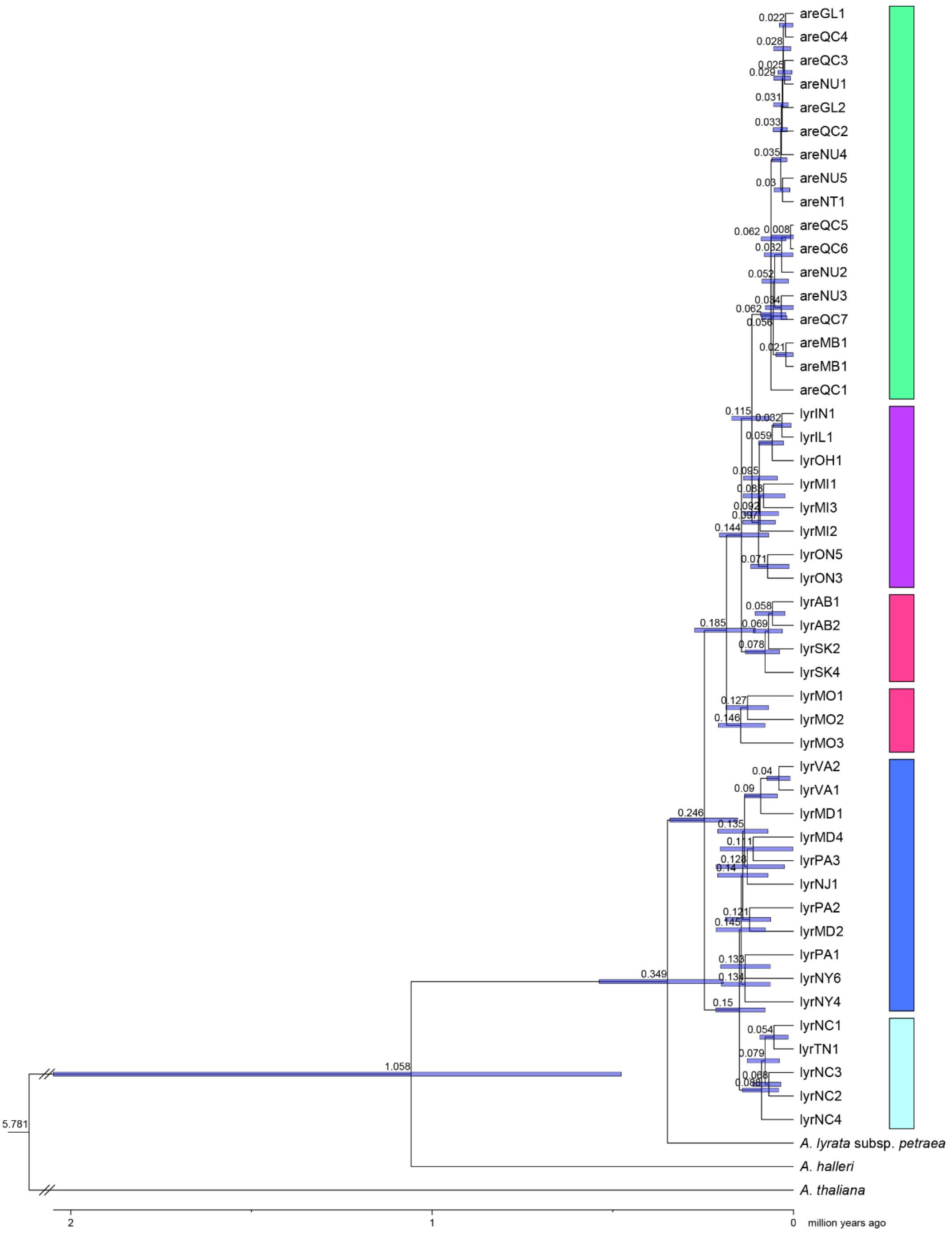

Supplementary Figure 3. Divergence time estimation based on the nuclear genome. Results are based on biallelic synonymous SNPs from the nuclear genome with a minimum distance of 100 kb. The SNAPP plugin for BEAST2 was used to estimate divergence times based on SNPs. Calibration was performed using the estimates obtained from the plastid divergence time estimation at three points: split of *A. halleri*, split of European *A. lyrata* subsp. *petraea*, and crown age of North American *A. lyrata* subsp. *lyrata* (abbreviated with lyr; are for *A. arenicola*). The combined results from eight independent runs of 100’000 MCMC generations, sampling every 100 generations and discarding the first 10% of each run as burn-in, are shown. Blue bars represent the 95% HPD intervals. ADMIXTURE results (K = 5) are shown on the right. Source data are provided as a Source Data file.

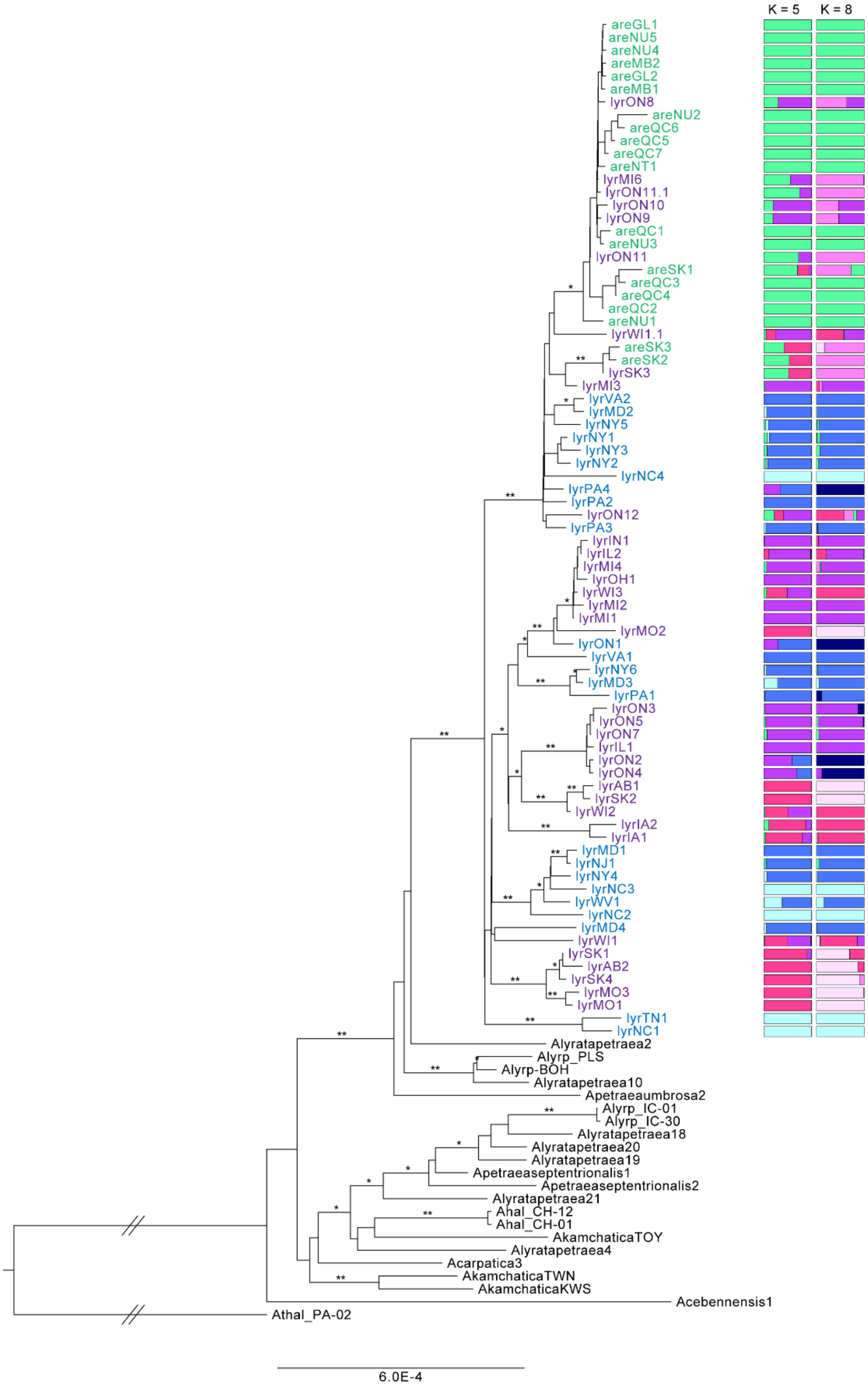

Supplementary Figure 4. Plastome maximum likelihood tree. Maximum likelihood reconstruction was conducted using RAxML. A reduced set of individuals, omitting identical sequences and selecting one individual per population, was used. *A. thaliana* was set as the outgroup. Population names are colored according to their origin for North American *Arabidopsis lyrata* (abbreviated with lyr; eastern cluster blue, western cluster purple) and *A. arenicola* (are; green). The assignment to K = 5 and K = 8 genetic clusters using ADMIXTURE analysis is given on the right. Bootstrap support from 1000 bootstrap replicates: ** 100; * 95-99. Source data are provided as a Source Data file.

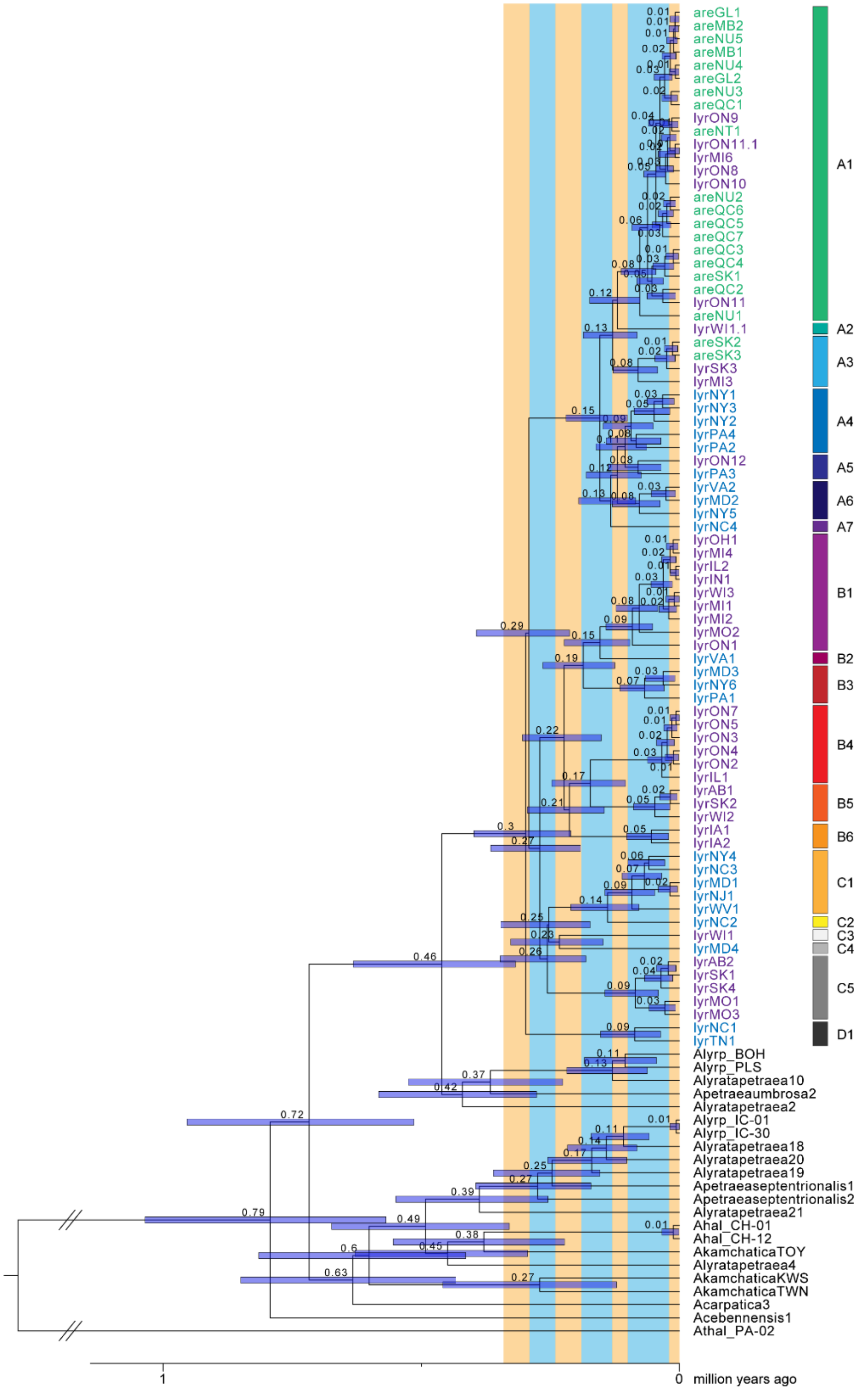

Supplementary Figure 5. Plastome divergence time estimates. A reduced set of individuals, omitting identical sequences and selecting one individual per population, was used. Secondary calibration was based on Novikova et al.[1] for four nodes: the root height (divergence of *A. thaliana* from the rest of the genus), the crown age of the remaining species, the crown age of *A. lyrata*, and the divergence between samples Aarenicola1 and Alyrl-14-ON4. Population names are colored according to their origin for North American *Arabidopsis lyrata* (abbreviated with lyr; eastern cluster blue, western cluster purple) and *A. arenicola* (are; green). Clades with an age of <150 ky are shown using the color bars on the right. Orange and blue background indicates interglacial and glacial periods of the past 340 ky, respectively. Source data are provided as a Source Data file.

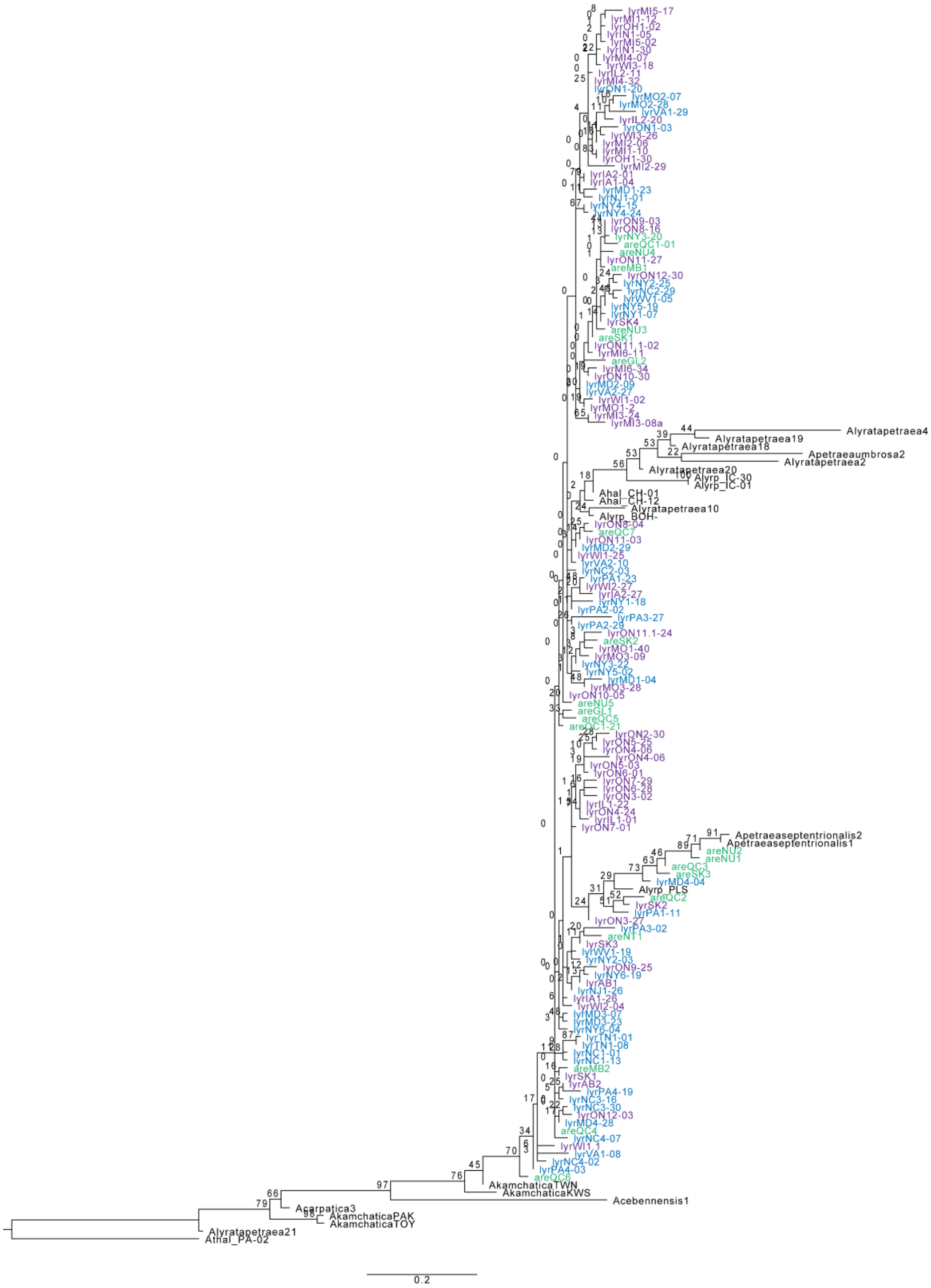

Supplementary Figure 6. Mitochondrial maximum likelihood tree. Maximum likelihood reconstruction was conducted using RAxML using only protein coding sequences from the mitochondrial genome. *A. thaliana* was set as the outgroup. Population names are colored according to their origin for North American *Arabidopsis lyrata* (abbreviated with lyr; eastern cluster blue, western cluster purple) and *A. arenicola* (are; green). Bootstrap support from 1000 bootstrap replicates is given (in %). The phylogenetic reconstruction based on protein coding regions from the mitochondrial genome only had very low bootstrap support at most branches. Using *A. thaliana* as an outgroup, the non-*A. lyrata* individuals were located at the more basal branches of the ML tree, with the exception of *A. halleri*, which grouped within the *A. lyrata* clade. Eurasian species from the *A. lyrata* species complex grouped within North American *A. lyrata*. However, bootstrap support was too low to draw any further conclusions from the mitochondrial dataset. Source data are provided as a Source Data file.

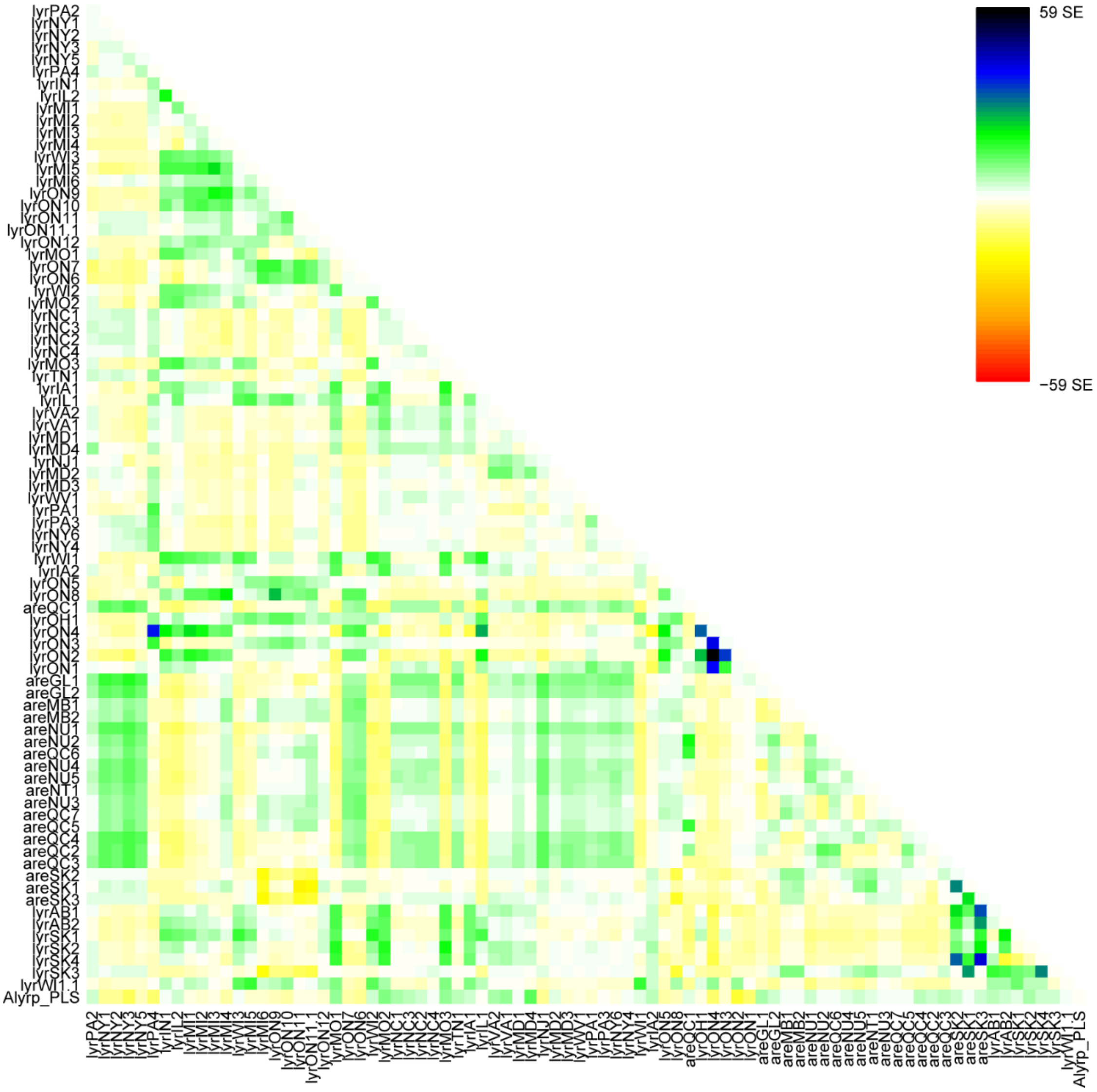

Supplementary Figure 7. TreeMix residuals for m = 0. Residuals are displayed in a heat map colored according to their standard error (SE), with colors corresponding to those given on the right side. Clusters of few outlier values indicate a bad fit of the model to the data. Source data are provided as a Source Data file.

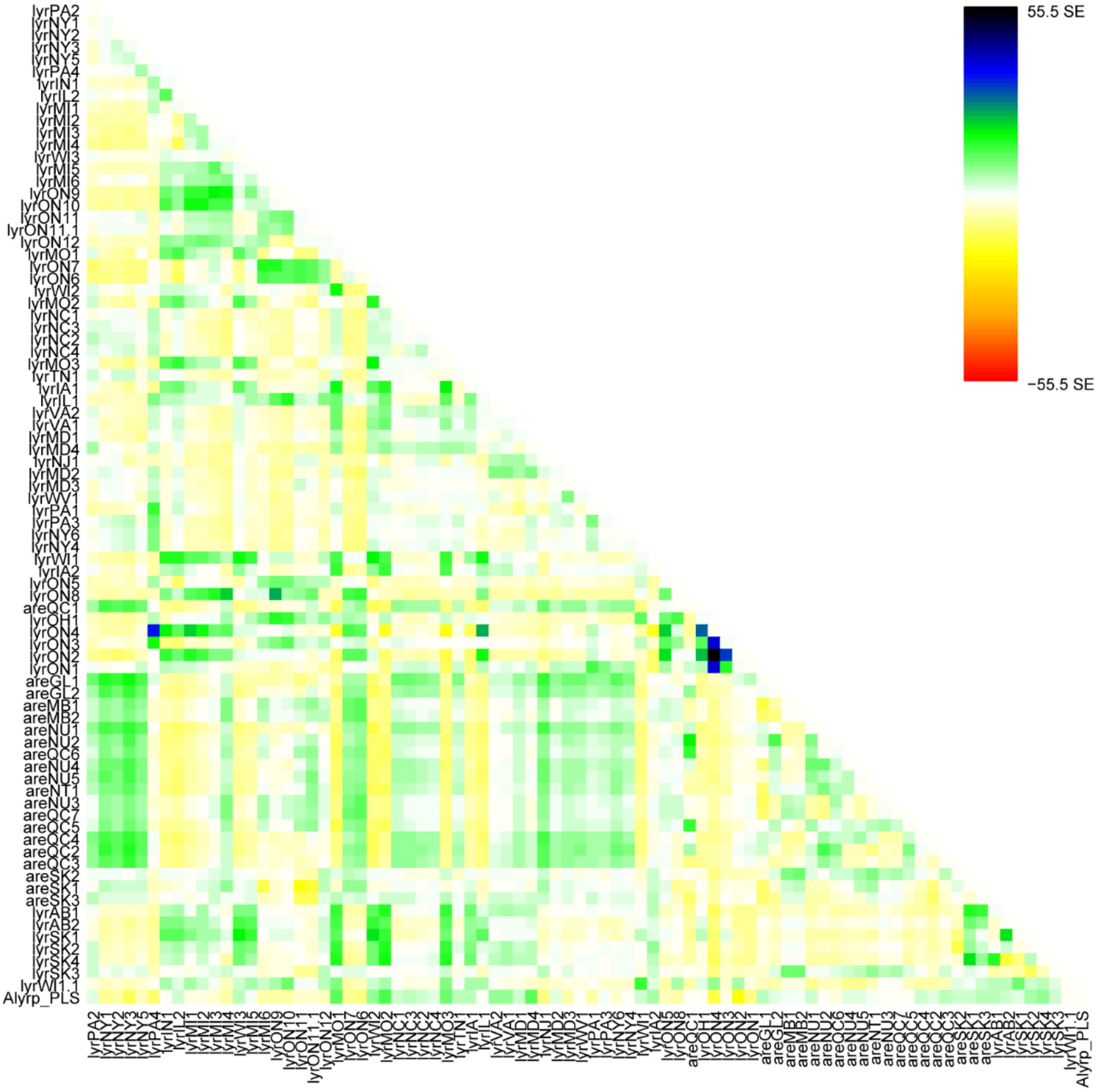

Supplementary Figure 8. TreeMix residuals for m = 1. Residuals are displayed in a heat map colored according to their standard error (SE), with colors corresponding to those given on the right side. Clusters of few outlier values indicate a bad fit of the model to the data. Source data are provided as a Source Data file.

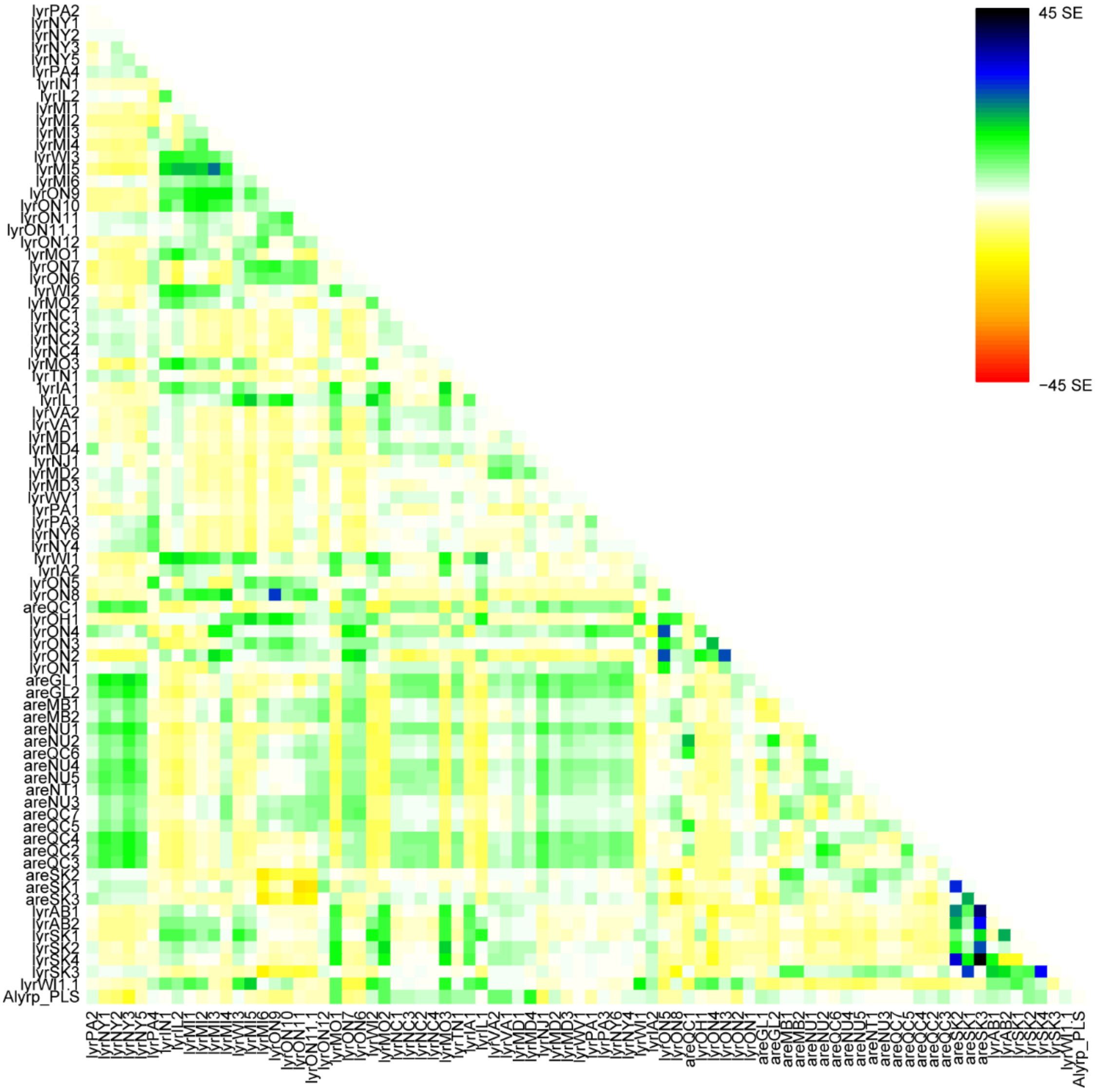

Supplementary Figure 9. TreeMix residuals for m = 2. Residuals are displayed in a heat map colored according to their standard error (SE), with colors corresponding to those given on the right side. Clusters of few outlier values indicate a bad fit of the model to the data. Source data are provided as a Source Data file.

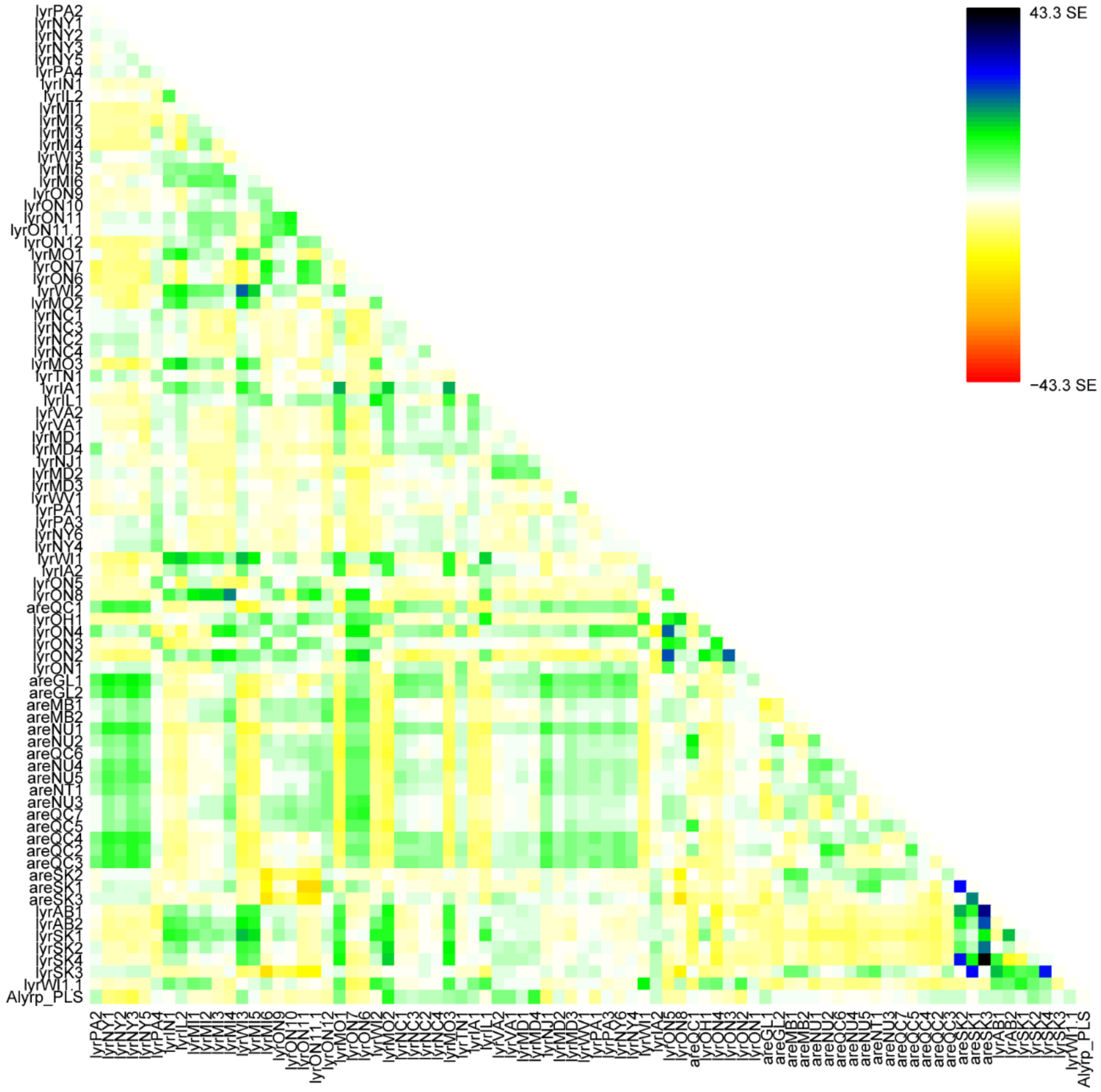

Supplementary Figure 10. TreeMix residuals for m = 3. Residuals are displayed in a heat map colored according to their standard error (SE), with colors corresponding to those given on the right side. Clusters of few outlier values indicate a bad fit of the model to the data. Source data are provided as a Source Data file.

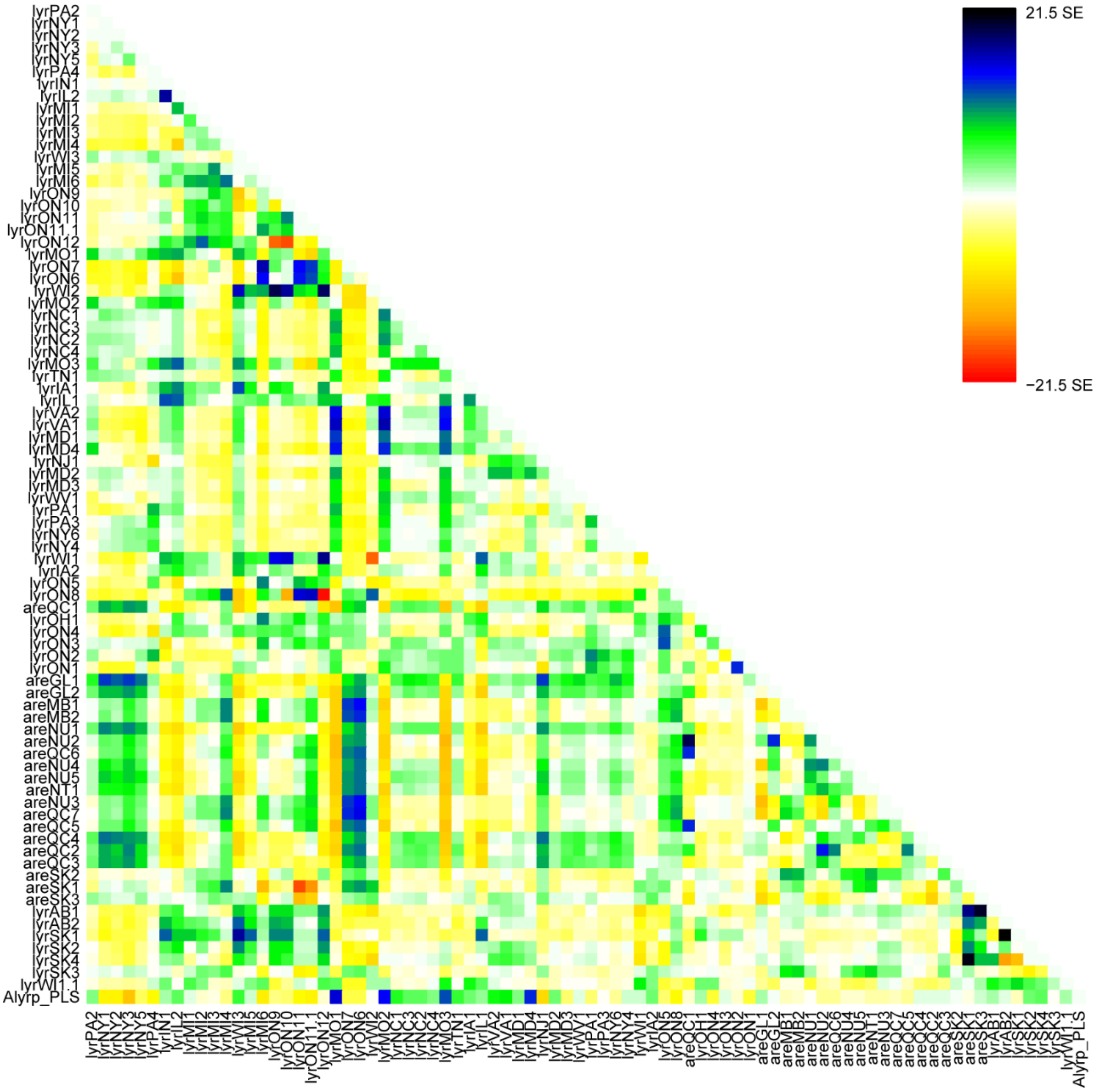

Supplementary Figure 11. TreeMix residuals for m = 4. Residuals are displayed in a heat map colored according to their standard error (SE), with colors corresponding to those given on the right side. The absence of few outlier values as well as the considerable jump in maximum SE value indicate a good fit of the model to the data. Source data are provided as a Source Data file.

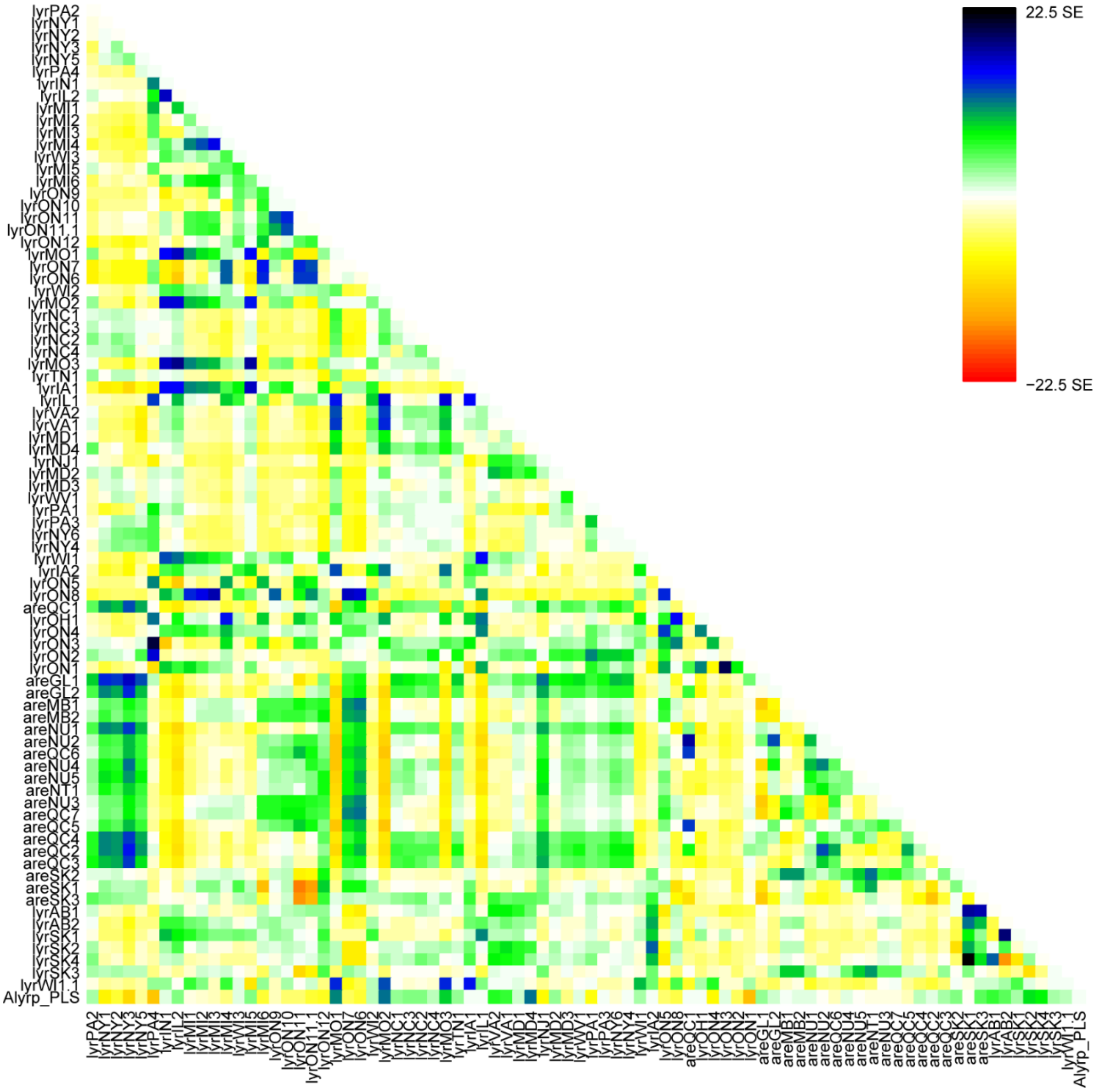

Supplementary Figure 12. TreeMix residuals for m = 5. Residuals are displayed in a heat map colored according to their standard error (SE), with colors corresponding to those given on the right side. The small decrease in maximum SE value indicates that the additional migration event does not increase the model fit considerably. Source data are provided as a Source Data file.

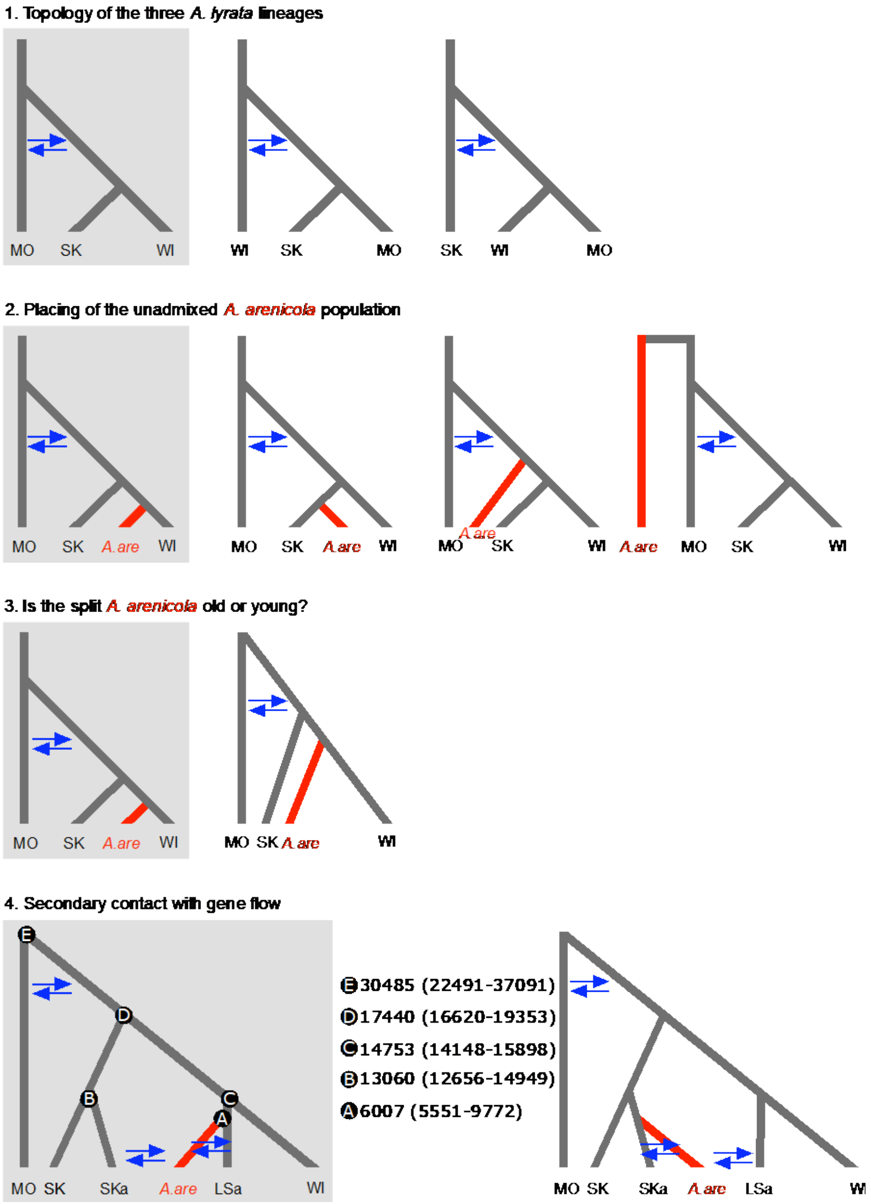

Supplementary Figure 13. Hierarchical demographic modelling comparing scenarios of the evolutionary history of *A. arenicola*. 1) We first established the backbone relationship of three western *A. lyrata* (super-)populations (samples considered in Supplementary Data 2) that had revealed no admixture with *A. arenicola* (Fig. 1d, e). The best supported model, highlighted in gray, indicates that the split between Missouri populations (MO) and populations in Saskatchewan (SK) and Wisconsin (WI) represents the oldest split. 2) Next, we placed *A. arenicola* (*A. are*), and the best supported model indicated its split from WI. 3) To test if the split between *A. arenicola* and WI occurred since or prior to the last glaciation period, we compared the two scenarios. The best fitting model implicates a recent split between *A. arenicola* form *A. lyrata*. 4) Finally, we included the two sets of populations that had shown admixture between *A. arenicola* and *A. lyrata* based on K = 5, Saskatchewan (SKa) and north shore Lake Superior (LSa) and compared two concurrent scenarios of speciation with gene flow and secondary contact. Here, a split of *A. arenicola* from population LSa followed by secondary contact with SKa was the better supporting model. For that last of the best models, split times and 95% CI are given. Details on model comparisons and estimates of the final best fitting model are given in Supplementary Data 3, 4. Source data are provided as Source Data files.

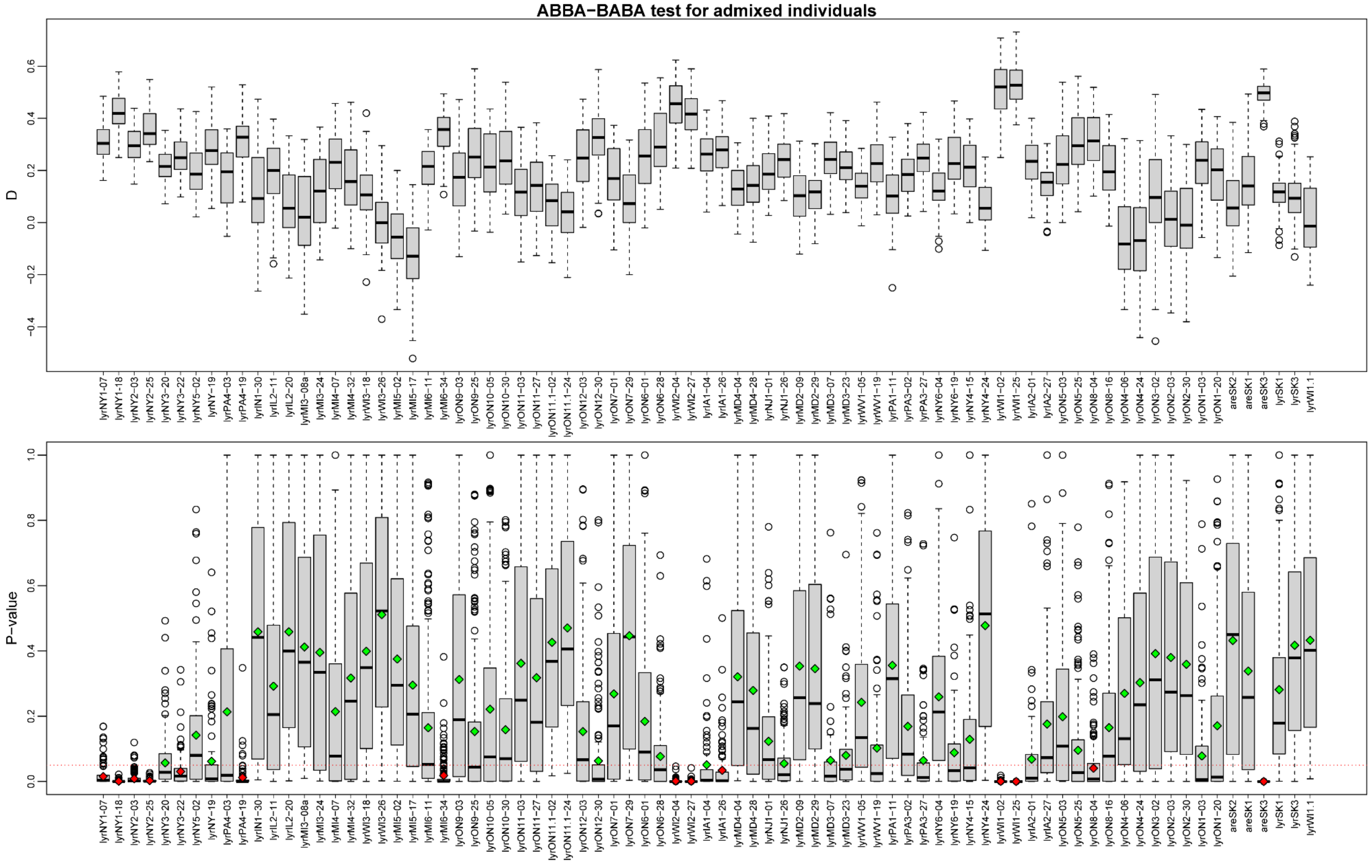

Supplementary Figure 14. Evidence for gene flow in admixed individuals based on cluster analysis. The panel on the top presents box plots of Patterson's *D*-statistic (top), the panel on the bottom box plots of P-values of individuals with evidence for admixture ≥10% under K = 5 (n = 89-180 depending on sample; box plot elements: center line is the median, box limits are the upper and lower quartiles, whiskers the 1.5x interquartile ranges and points the outliers). Samples with red (compared to green) dots on the bottom have a mean P < 0.05, indicating significant gene flow with the cluster contributing second in assignment in cluster analysis. Significance was tested based on 1000 bootstrap replicates. Source data are provided as a Source Data file.

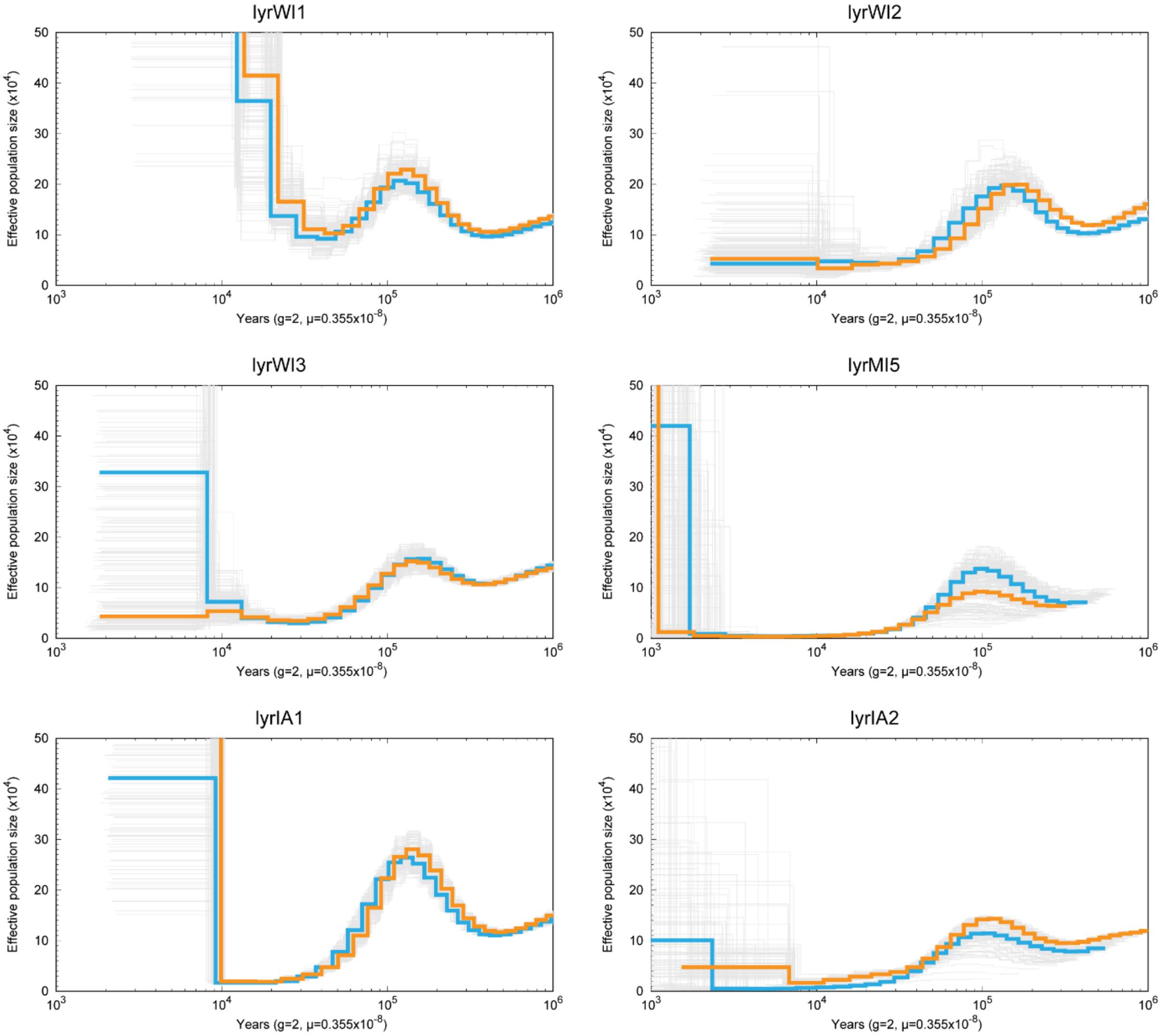

Supplementary Figure 15. Historic effective population size in populations in the Wisconsin cluster. Population size was estimated using a generation time of two years and mutation rate of 0.355 x 10^{-8}. Source data are provided as Source Data files.

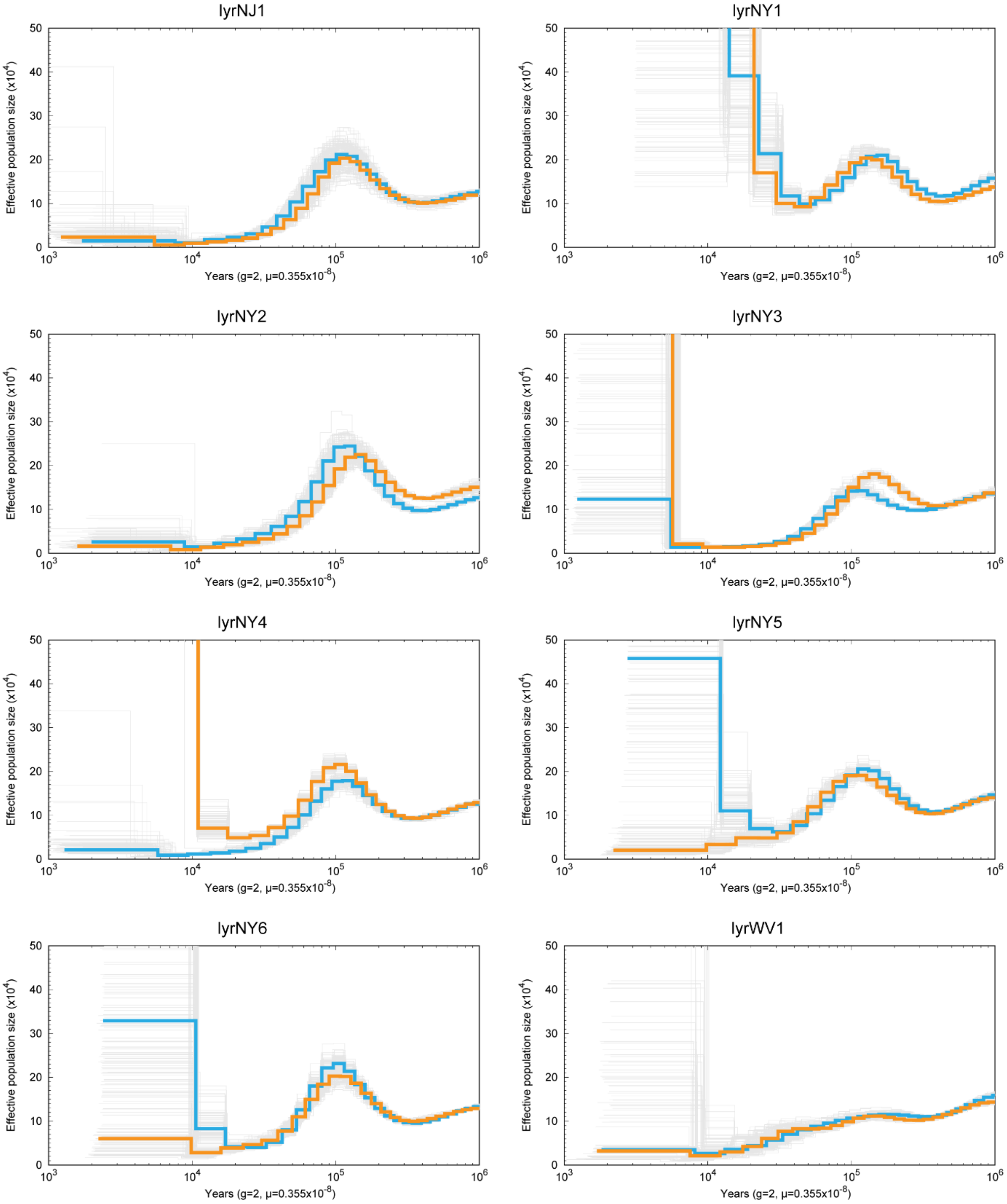

Supplementary Figure 16. Historic effective population size in populations in the Pennsylvania cluster (1/2). Population size was estimated using a generation time of two years and mutation rate of 0.355 x 10^{-8}. Source data are provided as Source Data files.

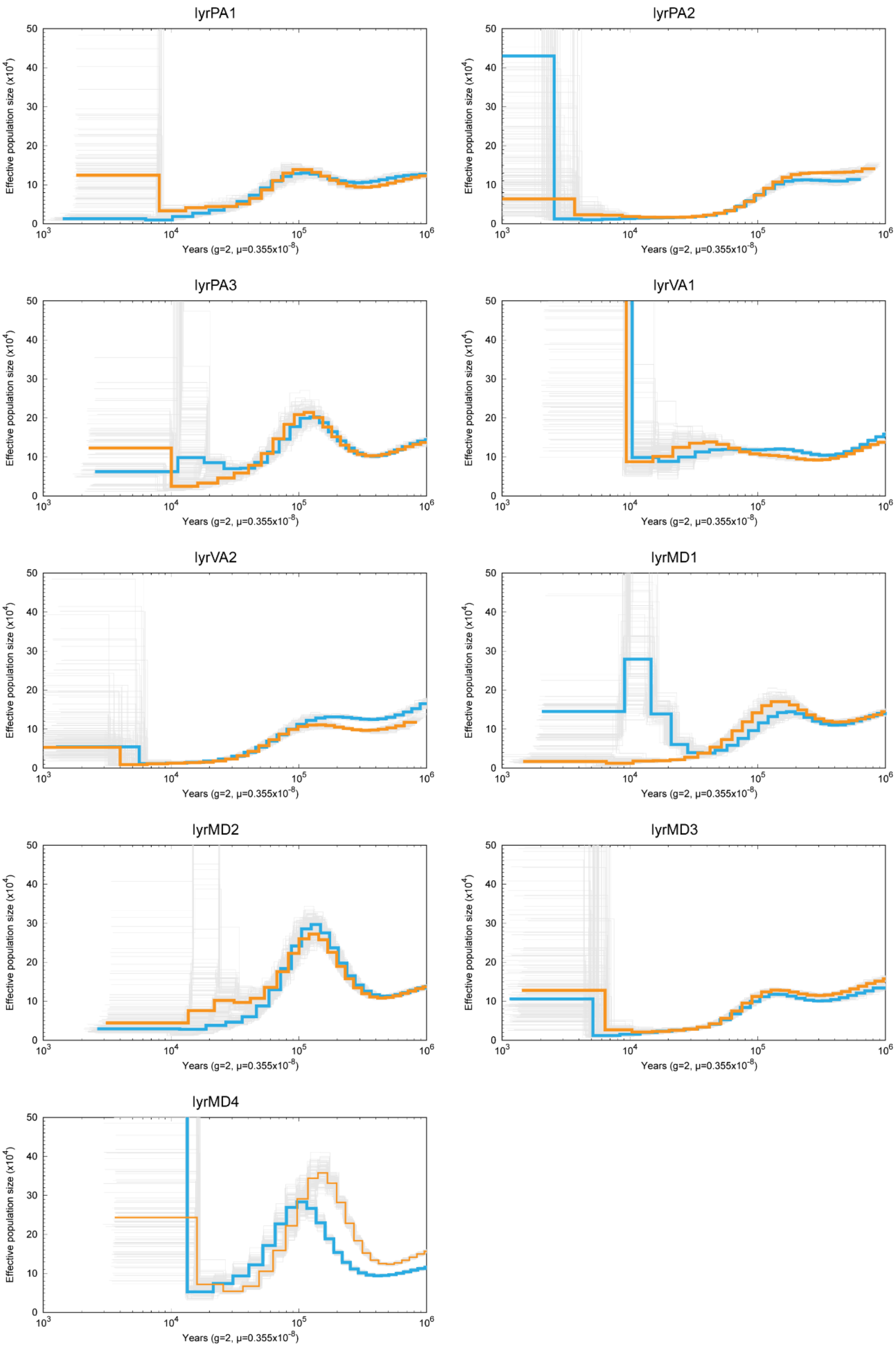

Supplementary Figure 17. Historic effective population size in populations in the Pennsylvania cluster (2/2). Population size was estimated using a generation time of two years and mutation rate of 0.355 x 10^{-8}. Source data are provided as Source Data files.

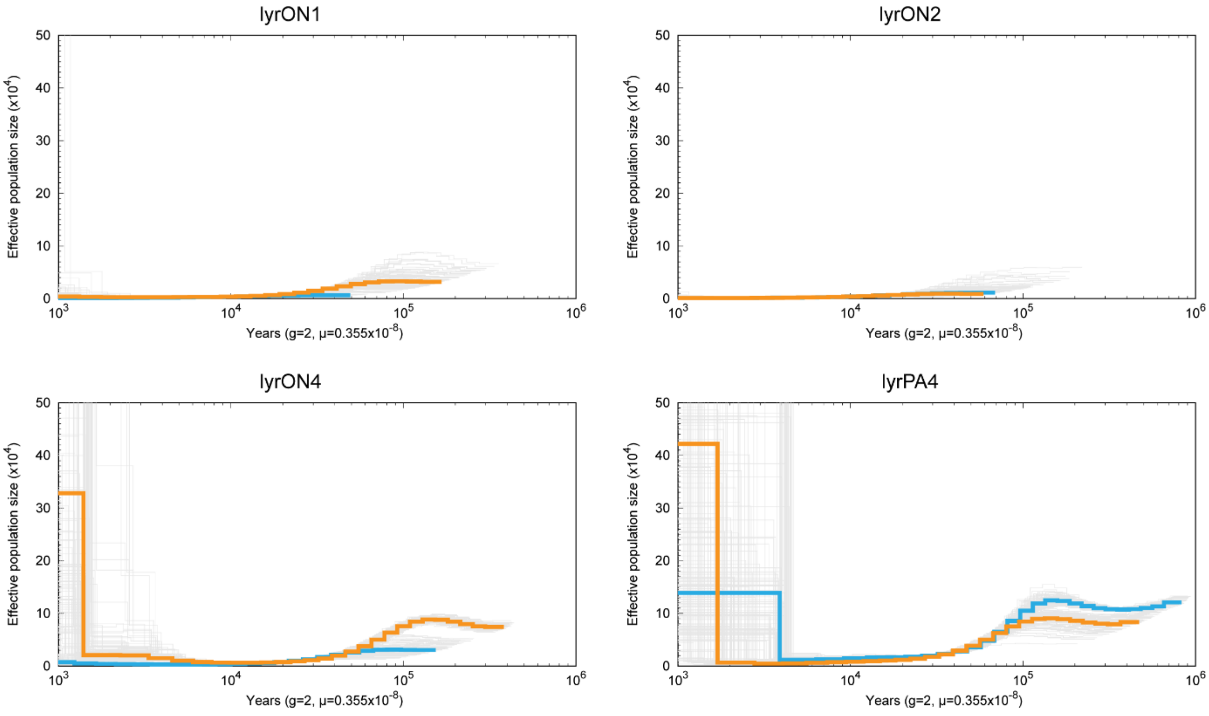

Supplementary Figure 18. Historic effective population size in populations around Lake Erie. Population size was estimated using a generation time of two years and mutation rate of 0.355 x 10^{-8}. Source data are provided as Source Data files.

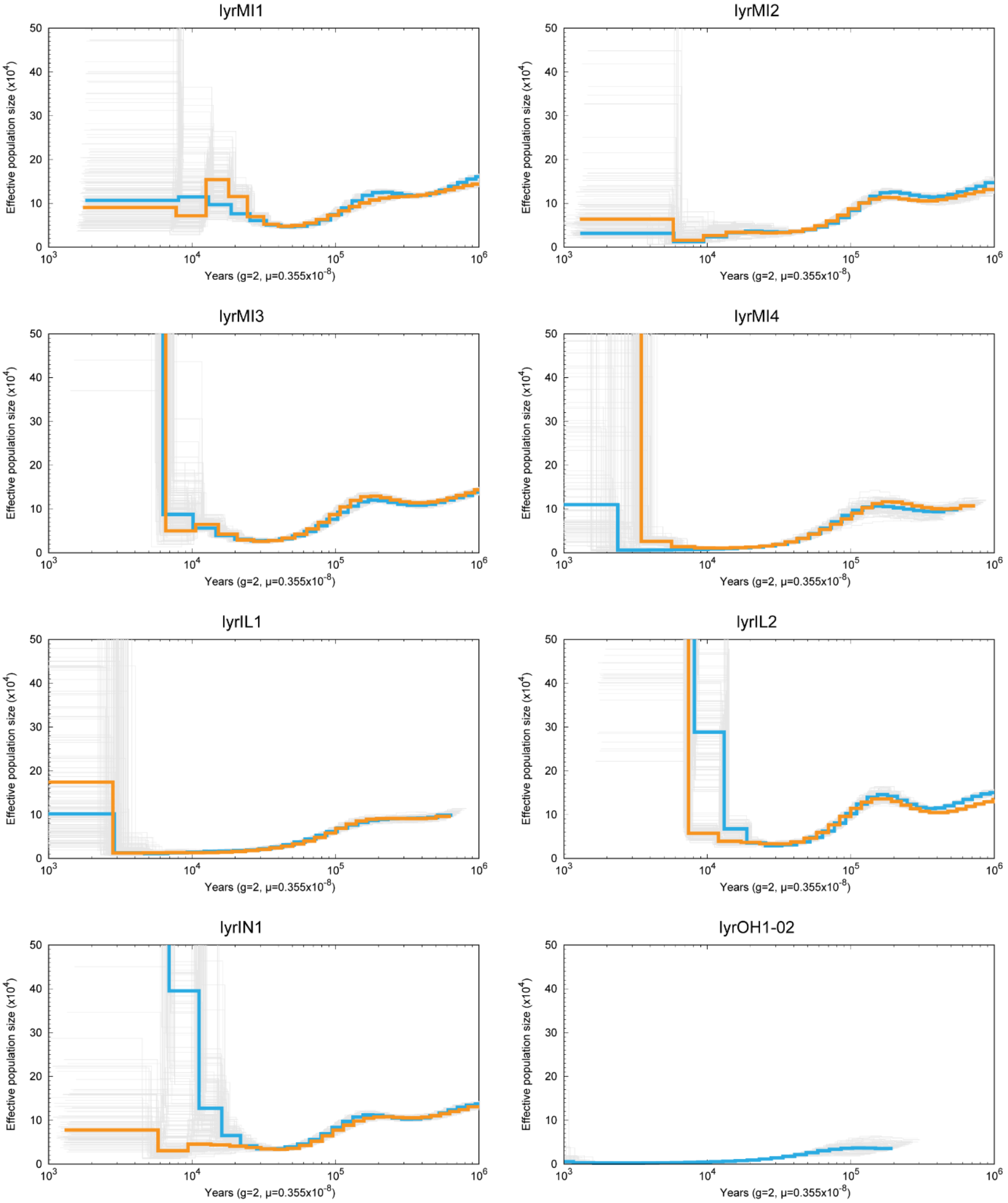

Supplementary Figure 19. Historic effective population size in populations in the Lake Michigan cluster (1/2). Population size was estimated using a generation time of two years and mutation rate of 0.355 x 10^{-8}. Source data are provided as Source Data files.

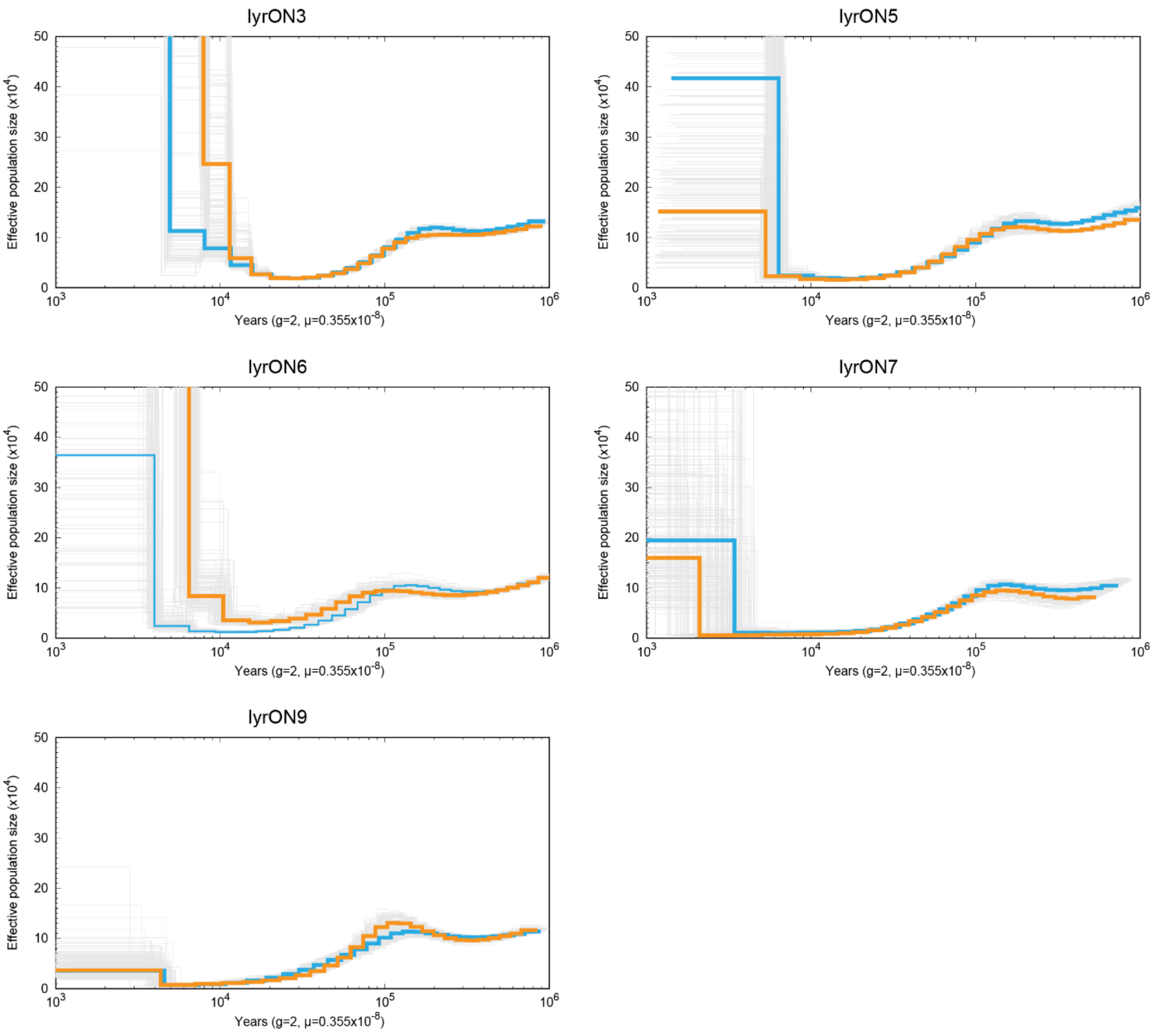

Supplementary Figure 20. Historic effective population size in populations in the Lake Michigan cluster (2/2). Population size was estimated using a generation time of two years and mutation rate of 0.355 x 10^{-8}. Source data are provided as Source Data files.

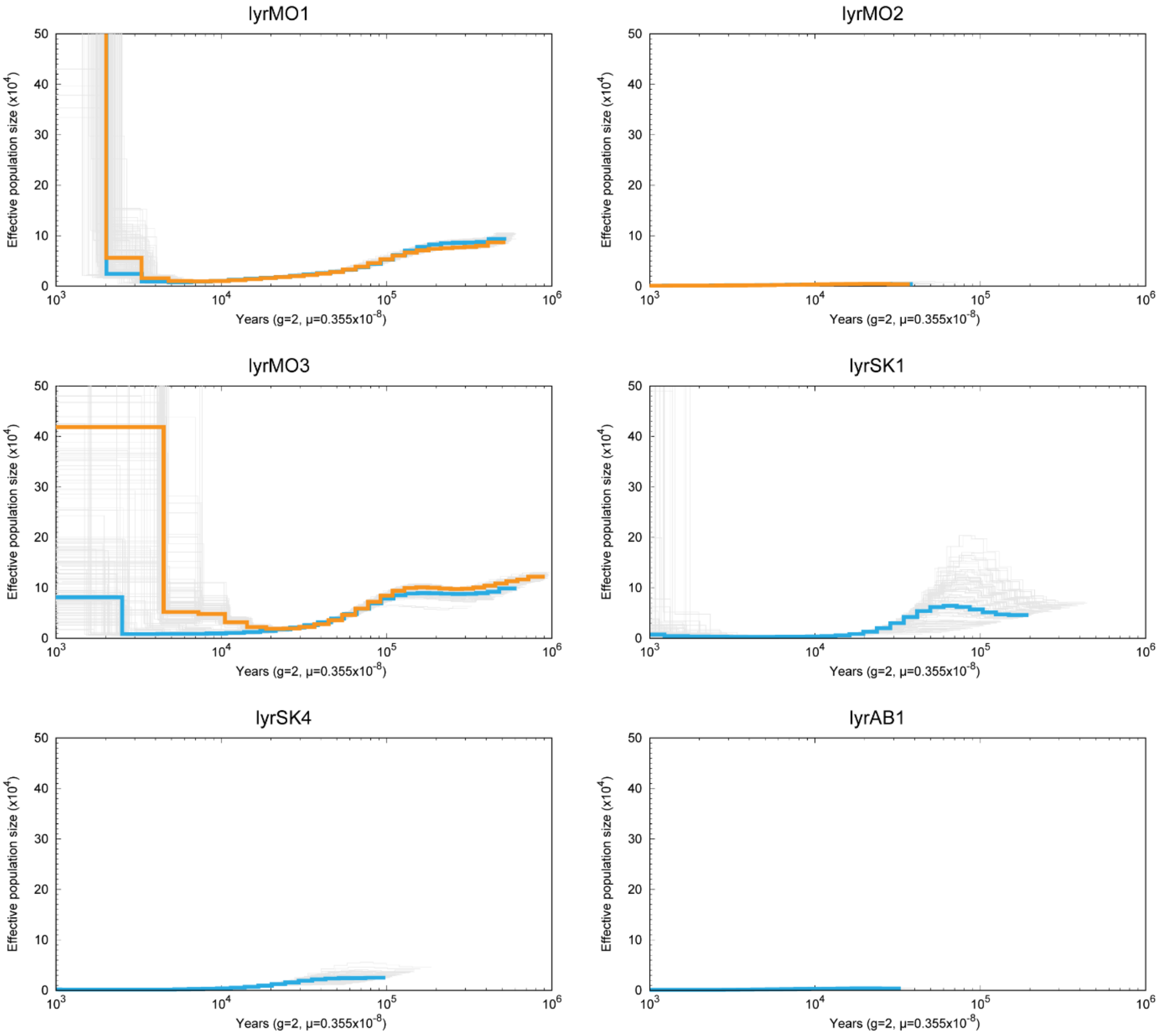

Supplementary Figure 21. Historic effective population size in populations in the Missouri-Saskatchewan cluster. Population size was estimated using a generation time of two years and mutation rate of 0.355 x 10^{-8}. Source data are provided as Source Data files.

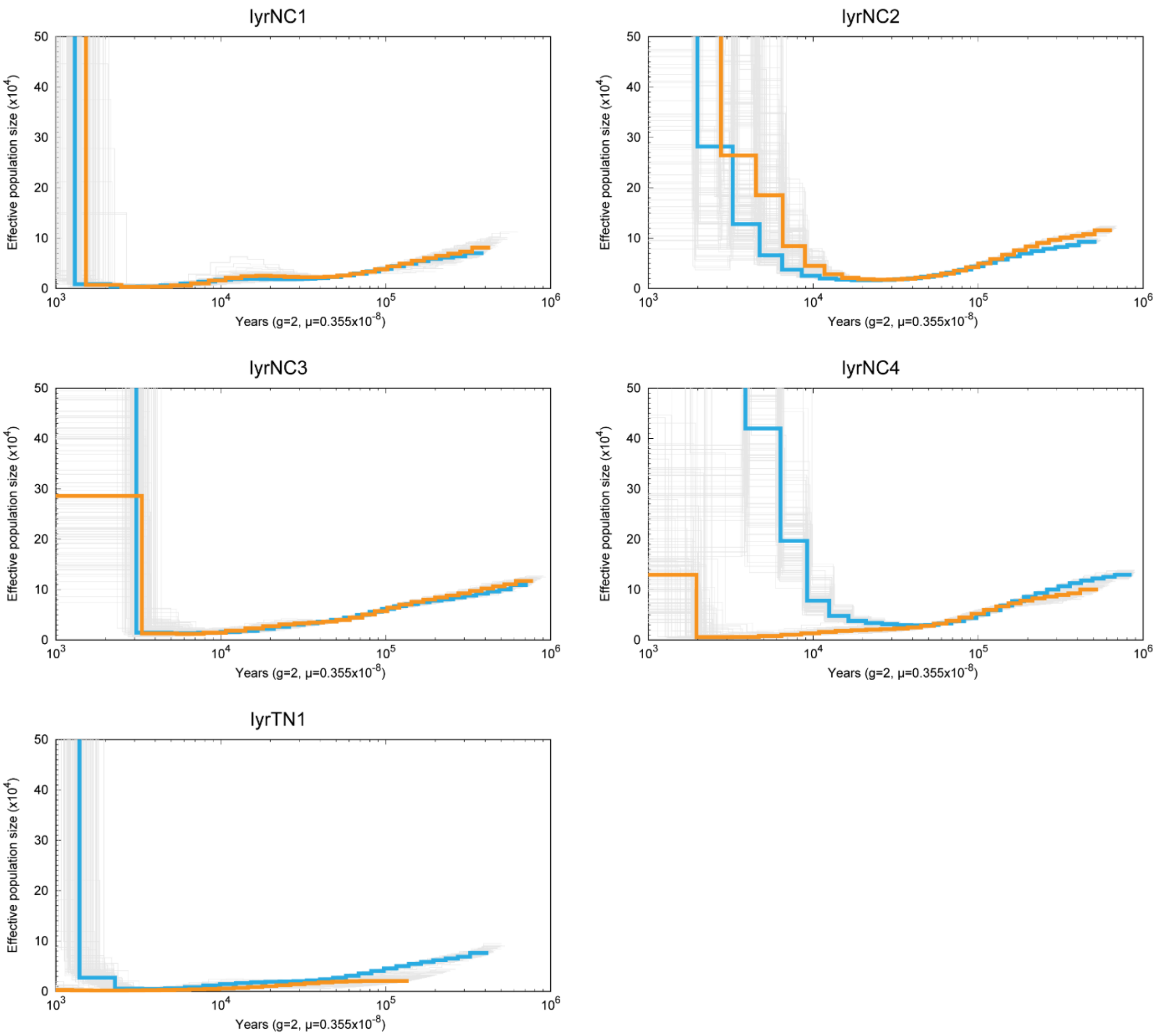

Supplementary Figure 22. Historic effective population size in populations in the North Carolina cluster. Population size was estimated using a generation time of two years and mutation rate of 0.355 x 10^{-8}. Source data are provided as Source Data files.

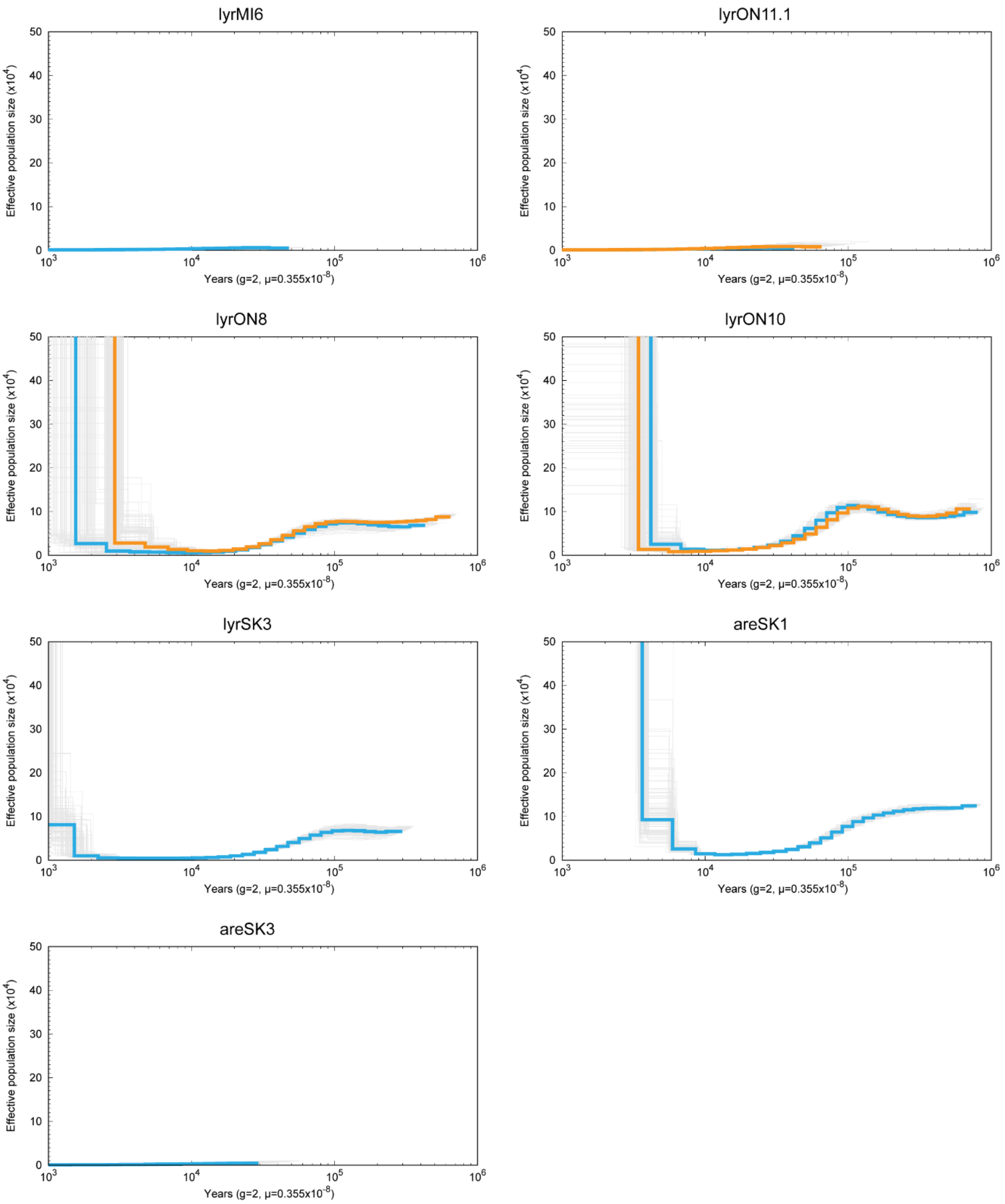

Supplementary Figure 23. Historic effective population size in populations in the *A. arenicola*-admixture cluster. Admixture clusters are described in the Supplementary Note (also see Fig. 1d). Population size was estimated using a generation time of two years and mutation rate of 0.355 x 10^{-8}. Source data are provided as Source Data files.

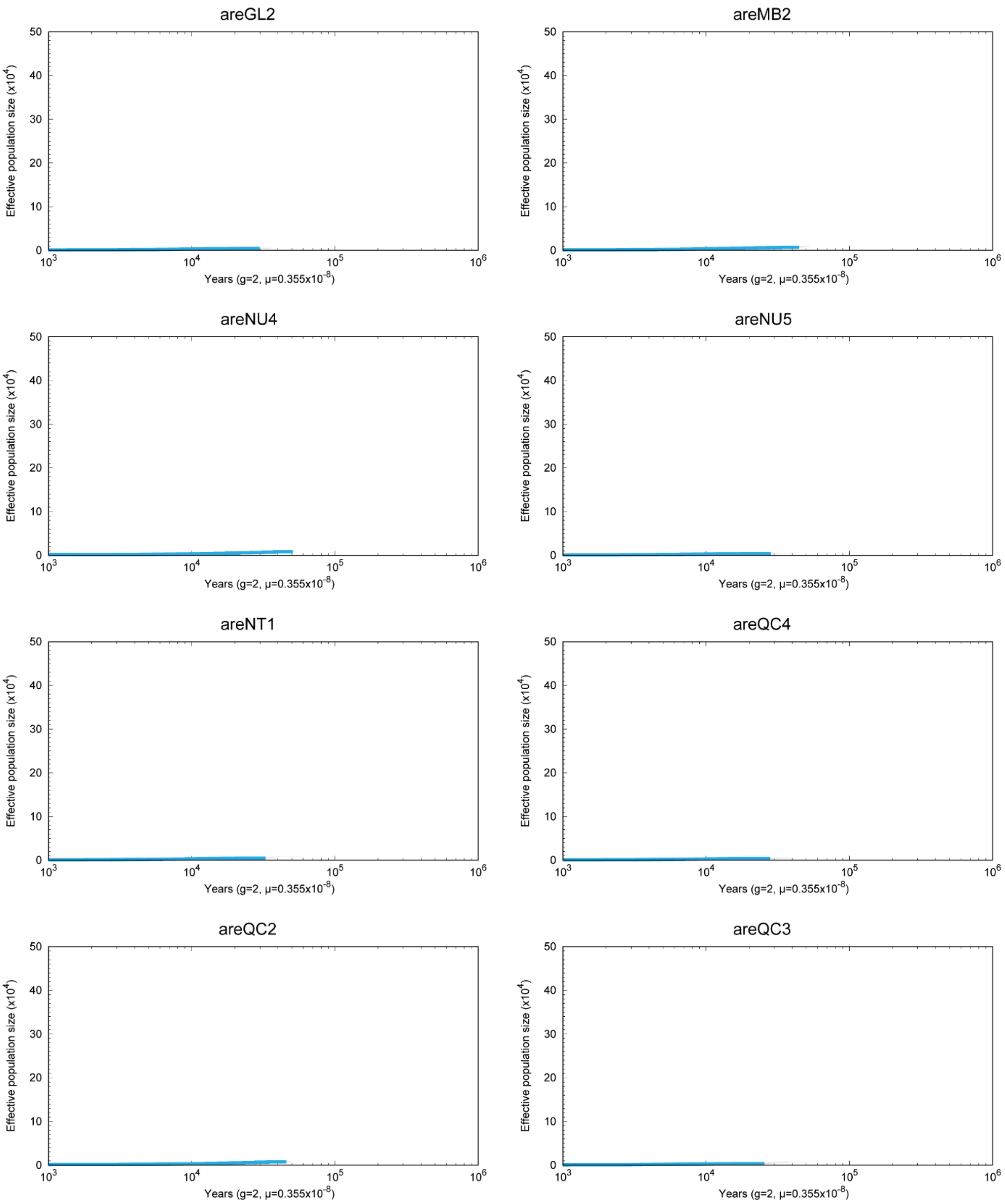

Supplementary Figure 24. Historic effective population size in populations of *A. arenicola*. Population size was estimated using a generation time of two years and mutation rate of 0.355 x 10^{-8}. Source data are provided as Source Data files.

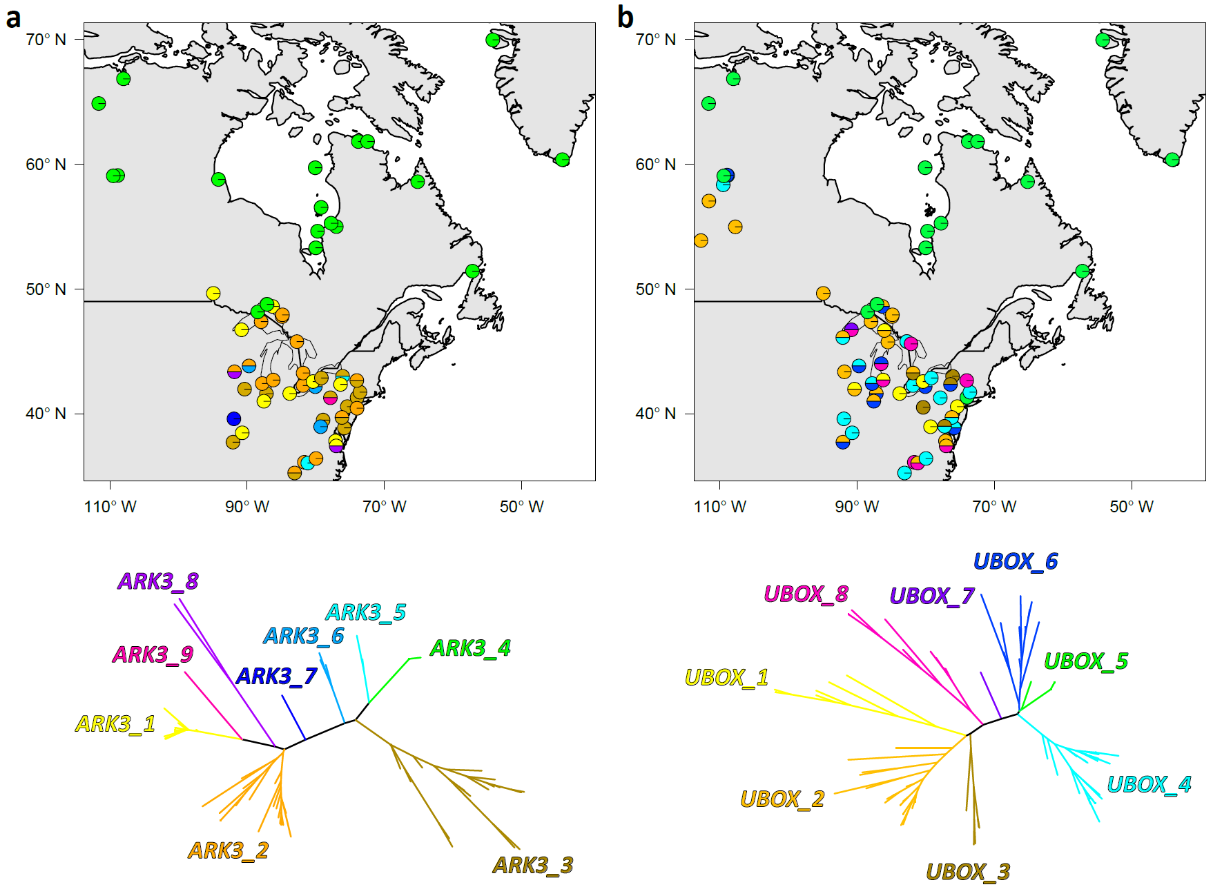

Supplementary Figure 25. Haplotype diversity in the genes flanking the self-incompatibility (S-)locus. Maps (on the top) show the distribution of the major haplotype groups for *ARK3* (a) and *UBOX* (b) that flank the S-locus. Gene-specific neighbor-joining trees (on the bottom) present the relationships among haplotype groups. For some individuals, haplotypes could only be reconstructed for some genes (Supplementary Data 6). Source data are provided as Source Data files.

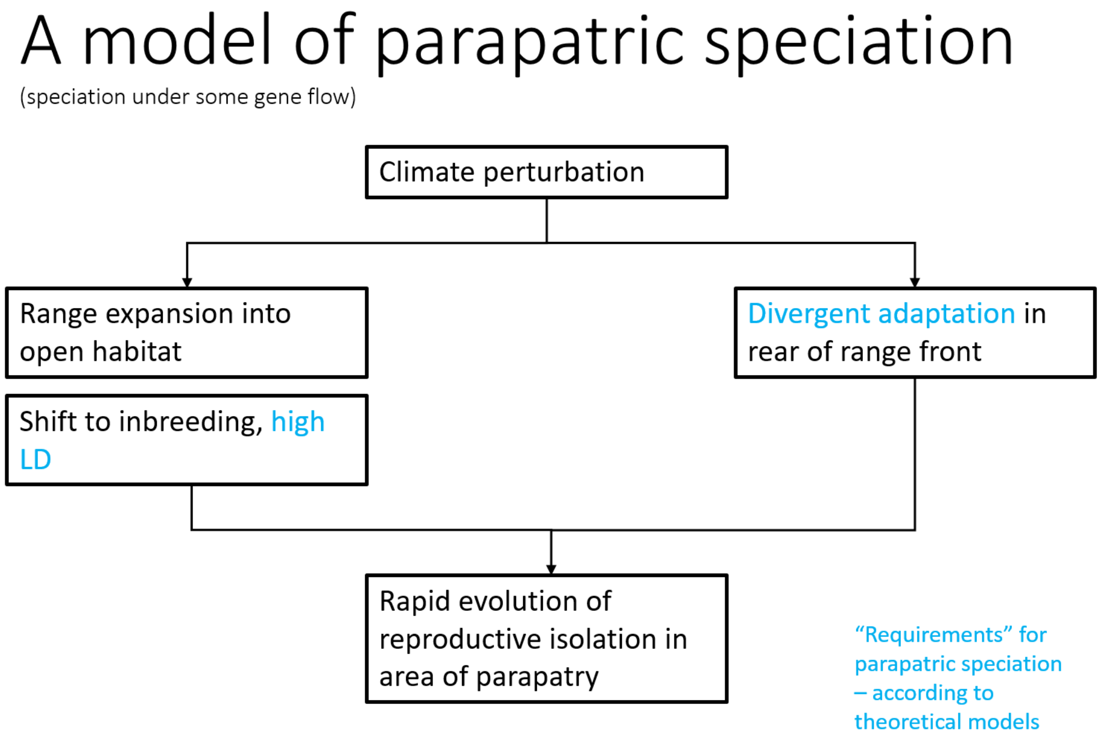

Supplementary Figure 26. A model of peripheral parapatric speciation driven by climate change, range expansion and a shift to inbreeding or selfing. Climate change can lead to the availability of open, suitable habitat into which a species may expand. Range expansion by serial bottlenecks may lead to (an evolutionary transition to) more inbreeding or selfing. As a consequence of more inbreeding, genome-wide LD increases. In parallel, in the rear of the expansion front, climate gradients may establish to which populations adapt divergently. Heightened LD and divergent adaptation are predicted to favor the evolution of reproductive isolation (reviewed by Kirkpatrick & Ravigné[32], *Am. Nat.* 159, S22–S35 [2002]) and contribute to peripheral parapatric speciation.

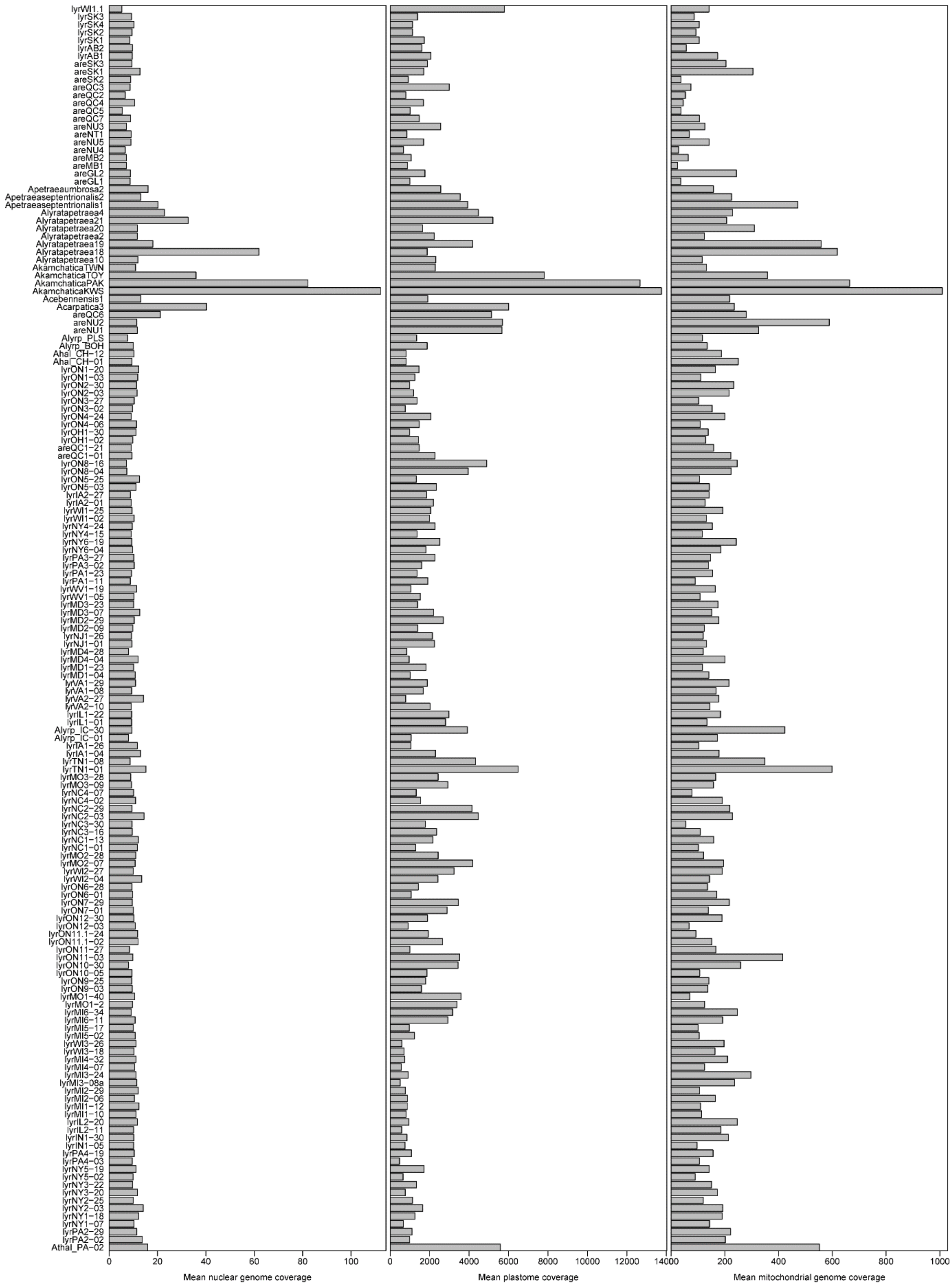

Supplementary Figure 27. Coverage statistics. Mean genome coverage for nuclear, plastid and mitochondrial genomes are given for every sample. North American *Arabidopsis lyrata* are abbreviated with lyr, *A. arenicola* with are. Source data are provided as a Source Data file.

Supplementary References

1. Novikova, P. Y. et al. Sequencing of the genus *Arabidopsis* identifies a complex history of nonbifurcating speciation and abundant trans-specific polymorphism. *Nature Genet.* **48**, 1077–1082 (2016).
2. Bolger, A. M., Lohse, M. & Usadel, B. Trimmomatic: a flexible trimmer for Illumina sequence data. *Bioinformatics* **30**, 2114–2120 (2014).
3. McKenna, A. et al. The genome analysis toolkit: a MapReduce framework for analyzing next-generation DNA sequencing data. *Genome Res.* **20**, 1297–1303 (2010).
4. Fracassetti, M., Griffin, P. C. & Willi, Y. Validation of pooled whole-genome re-sequencing in *Arabidopsis lyrata*. *PLoS ONE* **10**, e0140462 (2015).
5. Quinlan, A. R. BEDTools: the Swiss-army tool for genome feature analysis. *Curr. Protoc. Bioinformatics* **47**, 11.12.1–11.12.34 (2014).
6. Bryant, D., Bouckaert, R., Felsenstein, J., Rosenberg, N. A., RoyChoudhury, A. Inferring species trees directly from biallelic genetic markers: bypassing gene trees in a full coalescent analysis. *Mol. Biol. Evol.* **29**, 1917–1932 (2012).
7. Bouckaert, R. et al. BEAST 2: a software platform for bayesian evolutionary analysis (A Prlic, Ed.). *PLoS Comput. Biol.* **10**, e1003537 (2014).
8. Katoh, K. & Standley, D. M. MAFFT multiple sequence alignment software version 7: improvements in performance and usability. *Mol. Biol. Evol.* **30**, 772–780 (2013).
9. Castresana, J. Selection of conserved blocks from multiple alignments for their use in phylogenetic analysis. *Mol. Biol. Evol.* **17**, 540–552 (2000).
10. Stamatakis, A. RAxML version 8: a tool for phylogenetic analysis and post-analysis of large phylogenies. *Bioinformatics* **30**, 1312–1313 (2014).
11. Drummond, A. J., Suchard, M. A, Xie, D. & Rambaut, A. Bayesian phylogenetics with BEAUti and the BEAST 1.7. *Mol. Biol. Evol.* **29**, 1969–1973 (2012).
12. Delignette-Muller, M. L. & Dutang, C. Fitdistrplus: an R package for fitting distributions. *J. Stat. Softw.* **64**, 1–34 (2015).
13. Stadler, T. On incomplete sampling under birth–death models and connections to the sampling-based coalescent. *J. Theor. Biol.* **261**, 58–66 (2009).
14. Excoffier, L., Dupanloup, I., Huerta-Sánchez, E., Sousa, V. C. & Foll, M. Robust demographic inference from genomic and SNP data. *PLoS Genet.,* **9**, e1003905 (2013).
15. Marchi, N. et al. The genomic origins of the world's first farmers. *Cell* **185**, 1842–1859 (2022).
16. Ashikawa, I. Gene-associated CpG islands in plants as revealed by analyses of genomic sequences. *Plant J.* **26**, 617–625 (2001).
17. Haubold, B., Pfaffelhuber, P. & Lynch, M. mlRho - a program for estimating the population mutation and recombination rates from shotgun-sequenced diploid genomes. *Mol. Ecol.* **19 Suppl 1**, 277–284 (2010).

18. Lucek, K. & Willi, Y. Drivers of linkage disequilibrium across a species' geographic range. *PLoS Genet.* **17**, e1009477 (2021).

19. Watterson, G. A. On the number of segregating sites in genetical models without recombination. *Theor. Pop. Biol.* **7**, 256–276 (1975).

20. Ossowski, S. et al. The rate and molecular spectrum of spontaneous mutations in *Arabidopsis thaliana*. *Science* **327**, 92–94 (2010).

21. Griffin, P. C. & Willi, Y. Evolutionary shifts to self-fertilisation restricted to geographic range margins in North American *Arabidopsis lyrata*. *Ecol. Lett.* **17**, 484–490 (2014).

22. Durand, E. Y., Patterson, N., Reich, D. & Slatkin, M. Testing for ancient admixture between closely related populations. *Mol. Biol. Evol.* **28**, 2239–2252 (2011).

23. Blackmon, H. & Adams, R. H. EvobiR: comparative and population genetic analyses. R package version 1.1 (2015). https://CRAN.R-project.org/package=evobiR

24. Li, H. & Durbin, R. Inference of human population history from individual whole-genome sequences. *Nature* **475**, 493–496 (2011).

25. Li, H. A statistical framework for SNP calling, mutation discovery, association mapping and population genetical parameter estimation from sequencing data. *Bioinformatics* **27**, 2987–2993 (2011).

26. Pyhäjärvi, T., Aalto, E. & Savolainen, O. Time scales of divergence and speciation among natural populations and subspecies of *Arabidopsis lyrata* (Brassicaceae). *Am. J. Bot.* **99**, 1314–1322 (2012).

27. Ritland, K. Extensions of models for the estimation of mating systems using n independent loci. *Heredity* **88**, 221-228 (2002).

28. Genete, M., Castric, V. & Vekemans, X. Genotyping and de novo discovery of allelic variants at the Brassicaceae self-incompatibility locus from short-read sequencing data. *Mol. Biol. Evol.* **7**, 1193–1201 (2020).

29. Safonova, Y., Bankevich, A. & Pevzner, P. A. dipSPAdes: assembler for highly polymorphic diploid genomes. *J. Comput. Biol.* **22**, 528–545 (2015).

30. Noé, L. & Kucherov, G. YASS: enhancing the sensitivity of DNA similarity search. *Nucleic Acids Res.* **33**, W540–W543 (2005).

31. Lynch, M. et al. Genome-wide linkage-disequilibrium profiles from single individuals. *Genetics* **198**, 269–281 (2014).

32. Kirkpatrick, M. & Ravigné, V. Speciation by natural and sexual selection: models and experiments. *Am. Nat.* **159**, S22–S35 (2002).
