# Supplementary material for: Recent speciation associated with range expansion and a shift to self-fertilization in North American Arabidopsis
Source: Nat Commun. 2022 Dec 8;13:7564. doi: 10.1038/s41467-022-35368-1 (PMC9732334; doi:10.1038/s41467-022-35368-1)
Supplement: Supplementary file 2 — Description of Additional Supplementary Files [file 41467_2022_35368_MOESM2_ESM.pdf]

Description of Additional Supplementary Files

File Name: Supplementary Data 1
Description: **Sample information.**

File Name: Supplementary Data 2
Description: **Samples used for demographic modelling.**

File Name: Supplementary Data 3
Description: **Fit of demographic models to the observed data.** Given are log10 likelihood differences between observed and expected site-frequency spectra (ΔLL), Akaike information criterion (AIC) and difference in AIC (ΔAIC) between the best and all models for a given dataset.

File Name: Supplementary Data 4
Description: **Parameter estimates of the best supported model.** Revealed in step 4, (super-)population Aare splits from LSa and with secondary contact to SKa (see Supplementary Data 3).

File Name: Supplementary Data 5
Description: **Microsatellite genotypes.** Of three populations of Arabidopsis arenicola, one from western Quebec and two from Manitoba (areMBa: 58.78°N, 94.20°W; areMBb: 58.76°N, 93.95°W). For the population of western Quebec, offspring plants were genotyped for progeny array analysis. Missing values (-1) and non-amplified loci (.) are indicated. Primers were the same as those used in Griffin & Willi 2014 Ecology Letters doi: 10.1111/ele.12248.

File Name: Supplementary Data 6
Description: **S-locus haplotypes.** Overview of the presence (1) or absence (0) of haplotypes for the genes that either flank the S-locus (ARK3, UBOX) or are part of it (SRK).
