# Supplementary material for: Recent speciation associated with range expansion and a shift to self-fertilization in North American Arabidopsis
Source: Nat Commun. 2022 Dec 8;13:7564. doi: 10.1038/s41467-022-35368-1 (PMC9732334; doi:10.1038/s41467-022-35368-1)
Supplement: Supplementary file 4 — Reporting Summary [file 41467_2022_35368_MOESM4_ESM.pdf]

nature portfolio

Corresponding author(s): Yvonne Willi

Last updated by author(s): Nov 30, 2022

Reporting Summary

Nature Portfolio wishes to improve the reproducibility of the work that we publish. This form provides structure for consistency and transparency in reporting. For further information on Nature Portfolio policies, see our Editorial Policies and the Editorial Policy Checklist.

Statistics

For all statistical analyses, confirm that the following items are present in the figure legend, table legend, main text, or Methods section.

n/a	Confirmed	
☐	☒	The exact sample size (*n*) for each experimental group/condition, given as a discrete number and unit of measurement
☐	☒	A statement on whether measurements were taken from distinct samples or whether the same sample was measured repeatedly
☐	☒	The statistical test(s) used AND whether they are one- or two-sided<br>*Only common tests should be described solely by name; describe more complex techniques in the Methods section.*
☒	☐	A description of all covariates tested
☒	☐	A description of any assumptions or corrections, such as tests of normality and adjustment for multiple comparisons
☐	☒	A full description of the statistical parameters including central tendency (e.g. means) or other basic estimates (e.g. regression coefficient) AND variation (e.g. standard deviation) or associated estimates of uncertainty (e.g. confidence intervals)
☐	☒	For null hypothesis testing, the test statistic (e.g. *F*, *t*, *r*) with confidence intervals, effect sizes, degrees of freedom and *P* value noted<br>*Give P values as exact values whenever suitable.*
☐	☒	For Bayesian analysis, information on the choice of priors and Markov chain Monte Carlo settings
☐	☒	For hierarchical and complex designs, identification of the appropriate level for tests and full reporting of outcomes
☒	☐	Estimates of effect sizes (e.g. Cohen's *d*, Pearson's *r*), indicating how they were calculated

Our web collection on statistics for biologists contains articles on many of the points above.

Software and code

Policy information about availability of computer code

Data collection	*Provide a description of all commercial, open source and custom code used to collect the data in this study, specifying the version used OR state that no software was used.*
Data analysis	From raw sequences to annotated SNPs: trimmomatic v. 0.36; bwa v. 0.7.13; SAMtools v. 1.3.1; picard v. 2.8.0; GATK v. 3.7; bedtools; SnpEff. Phylogeography: ADMIXTURE v. 1.3.0; VCFtools; RAxML v. 8.2.8; SNAPP; BEAST v. 2.6.2 and 1.8.3; FigTree v. 1.4.4; MAFFT v. 7.164; Gblocks v. 0.91; R package fitdistrplus; TreeMix v. 1.13. Demographic analyses: fastsimcoal2 v. 2.6; easySFS; mlRho v. 2.9; R package evobiR; PSMC; bcftools; MLTR; SPAdes v. 3.10.1; YASS v. 1.14. Figures: R v. 4.1.2; R packages akima, ape, fields, gdalUtils, grDevices, lmomco, mapdata, mapplots, maps, maptools, phytools, plotrix, PBSmapping, rgdal, rgeos, and sp.

For manuscripts utilizing custom algorithms or software that are central to the research but not yet described in published literature, software must be made available to editors and reviewers. We strongly encourage code deposition in a community repository (e.g. GitHub). See the Nature Portfolio guidelines for submitting code & software for further information.

Data

Policy information about availability of data

All manuscripts must include a data availability statement. This statement should provide the following information, where applicable:

- Accession codes, unique identifiers, or web links for publicly available datasets
- A description of any restrictions on data availability
- For clinical datasets or third party data, please ensure that the statement adheres to our policy

Raw sequences of newly sequenced individual plants are available under the ENA/GenBank projects PRJEB30473 [https://www.ncbi.nlm.nih.gov/bioproject/PRJEB30473] and PRJEB23202 [https://www.ncbi.nlm.nih.gov/bioproject/?term=PRJEB23202] (see Supplementary Table Data 1). Sequences of further plants were

accessed from ENA/GenBank (see Supplementary Table Data 1). Source data are provided with this paper.

Field-specific reporting

Please select the one below that is the best fit for your research. If you are not sure, read the appropriate sections before making your selection.

- [ ] Life sciences
- [ ] Behavioural & social sciences
- [x] Ecological, evolutionary & environmental sciences

For a reference copy of the document with all sections, see nature.com/documents/nr-reporting-summary-flat.pdf

Ecological, evolutionary & environmental sciences study design

All studies must disclose on these points even when the disclosure is negative.

Study description	An evolutionary genomics study on the biogeographic and demographic history of a newly emerging species by peripheral parapatric speciation in the genus Arabidopsis
Research sample	Sequence analyses on populations from across the ranges of Arabidopsis lyrata subsp. lyrata and Arabidopsis arenicola
Sampling strategy	Populations sampled to cover the ranges of Arabidopsis lyrata subsp. lyrata and Arabidopsis arenicola, with 61 and 20 populations, respectively. 1-2 samples of each population, either newly collected in the field, or from herbaria
Data collection	Localization of each sample with a GPS tracker in the field or location information of each herbarium specimen
Timing and spatial scale	Field sampling of Arabidopsis lyrata subsp. lyrata occurred between 2007 and 2014
Data exclusions	NA
Reproducibility	Replication was provided by relatively dense sampling of populations in the field, and by collecting mostly 2 specimen per population.
Randomization	NA
Blinding	NA

Did the study involve field work? [ ] Yes [x] No

Reporting for specific materials, systems and methods

We require information from authors about some types of materials, experimental systems and methods used in many studies. Here, indicate whether each material, system or method listed is relevant to your study. If you are not sure if a list item applies to your research, read the appropriate section before selecting a response.

Materials & experimental systems

n/a	Involved in the study
[x]	[ ] Antibodies
[x]	[ ] Eukaryotic cell lines
[x]	[ ] Palaeontology and archaeology
[x]	[ ] Animals and other organisms
[x]	[ ] Human research participants
[x]	[ ] Clinical data
[x]	[ ] Dual use research of concern

Methods

n/a	Involved in the study
[x]	[ ] ChIP-seq
[x]	[ ] Flow cytometry
[x]	[ ] MRI-based neuroimaging
